# Synergistic action between peptide-neomycin conjugates and polymyxin B against multidrug-resistant gram-negative pathogens

**DOI:** 10.3389/fmicb.2025.1605813

**Published:** 2025-08-07

**Authors:** Sandra Story, Liuwei Jiang, Alain S. Leutou, Dev P. Arya

**Affiliations:** ^1^NUBAD, LLC, Greenville, SC, United States; ^2^Laboratory of Medicinal Chemistry, Department of Chemistry, Clemson University, Clemson, SC, United States

**Keywords:** aminoglycosides, peptide neomycin conjugates, polymyxins, synergy, drug-resistant bacteria, amino acids

## Abstract

Globally, it is predicted that by 2050, 10 million people will die annually because of infections with drug-resistant bacteria. Since antibacterial agents with novel mechanisms of action have not been developed in the past 30 years, there has been a surge of interest in combination therapies using existing drugs. The combination of aminoglycosides and colistin is often used to treat pneumonia caused by multidrug-resistant bacteria. The goal of this study is to investigate the relationship between the antibacterial activity of a peptide-neomycin library and polymyxin B in extensively drug-resistant and pandrug-resistant bacteria. The peptide-neomycin library contained conjugates with one or two amino acids linked to neomycin, rendering them unsuitable substrates for aminoglycoside-modifying enzymes. Neomycin- susceptible and neomycin-resistant members of *Acinetobacter baumannii, Klebsiella pneumoniae*, and *Pseudomonas aeruginosa* were screened for synergy with polymyxin B using two-way checkerboard and time-kill methods. Most *A. baumannii* strains are resistant to amikacin, gentamicin, tobramycin, and plazomicin, and approximately half are susceptible to neomycin. *P. aeruginosa* strains have a similar resistance profile but was more susceptible to plazomicin. *K. pneumoniae* strains are most susceptible to a wide variety of aminoglycosides. Bacteria challenged with a combination of neomycin, other aminoglycosides, and polymyxin B exhibited an additive to indifferent relationship, whereas synergy was found with several neomycin-peptide conjugates containing cysteine, arginine, or tryptophan, lowering the minimal inhibitory concentration for the peptide-neomycin conjugate by 8-64-fold and polymyxin B by 2-8-fold. Cysteine, arginine, or tryptophan conjugates were the most effective against *A. baumannii* and *K. pneumoniae* carrying a 16S rRNA methyltransferase gene and a pandrug-resistant *P. aeruginosa* strain. Resistance to the combination of R-, C-, or RC-NEO conjugates and PB did not develop over a 14-day period in neomycin-susceptible strains of *A. baumannii, K. pneumoniae*, and *P. aeruginosa*. Based on this survey of the peptide-neomycin library, circumvention of aminoglycoside-modifying enzymes and alluding to bacterial resistance is an important step toward the design and development of peptide aminoglycoside-based motifs for antimicrobial drug development.

## Graphical Abstract

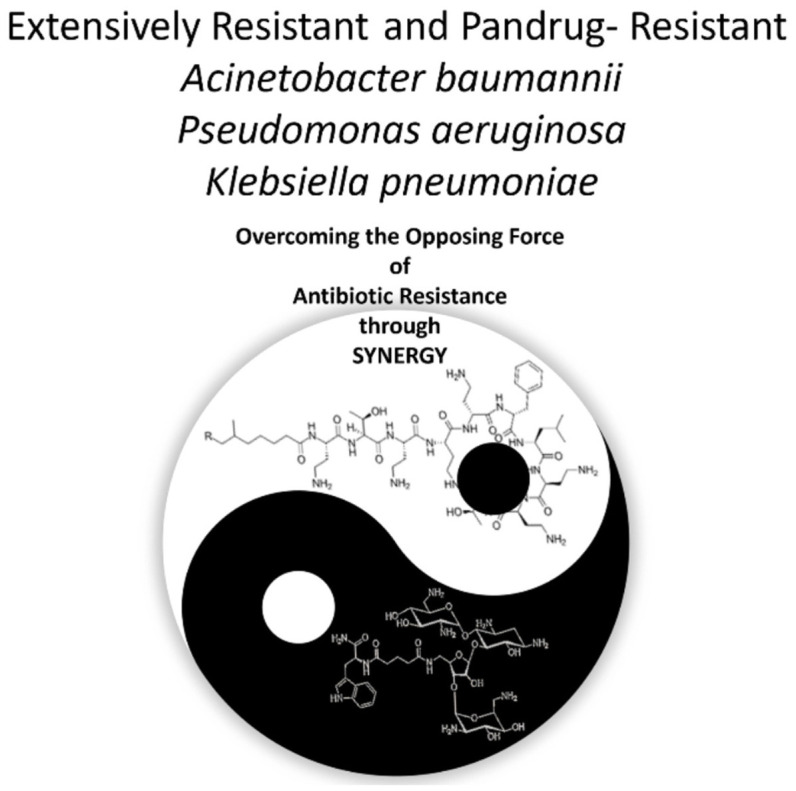

## 1 Introduction

The global burden associated with antibacterial resistance has resulted in an estimated 4.71 million deaths reported for 2021 (Murray et al., 2022; World Health Organization, 2024; Naghavi et al., 2024). The World Health Organization's (WHO) most recent update of the list of pathogens in rank order of critical and high-priority pathogens includes, carbapenem-resistant *Klebsiella pneumoniae*, third-generation cephalosporin-resistant *Escherichia coli*, carbapenem-resistant *Acinetobacter baumannii*, rifampicin-resistant *Mycobacterium tuberculosis* fluoroquinolone-resistant *Salmonella* Typhi, fluoroquinolone-resistant *Shigella* spp., vancomycin-resistant *Enterococcus faecium*, and carbapenem-resistant *Pseudomonas aeruginosa* (Sati et al., 2025). The natural habitats of *A. baumannii, Mycobacterium* spp., and *P. aeruginosa* are soil and aquatic environments, whereas *E. coli, K. pneumoniae*, and other members of the order Enterobacterales are part of the normal human intestinal microbial flora. These opportunistic pathogens comprise the *ESKAPE* opportunistic pathogens, including *Enterococcus faecium, Staphylococcus aureus, Klebsiella pneumoniae, Acinetobacter baumannii, Pseudomonas aeruginosa*, and *Enterobacter* spp., which are identified as being highly virulent and antibiotic resistant and are the leading cause of hospital-acquired infections (De Oliveira et al., 2020). In particular, *A. baumannii* has been identified as having high rates of acquired pandrug resistance, defined as resistance to all agents within all classes of antibiotics (Falagas and Bliziotis, 2007), because of its versatility in the upregulation of intrinsic resistance determinants and the rapid acquisition of multiple antibiotic resistance genes under the selective pressure of antibiotic use. Among the well-characterized antibiotic resistance mechanisms, aminoglycoside-modifying enzymes (AMEs) are the most abundant and diverse in nature, with >85 resistance determinants identified (Ramirez and Tolmasky, 2010, 2017; Aishwarya et al., 2020; Fernanda et al., 2022). Aminoglycoside resistance mechanisms fall into three categories: (1) enzymatic modification of antibiotics by AMEs (acquired resistance), (2) alteration or upregulation of intrinsic factors (outer membrane proteins, efflux pumps, and penicillin-binding proteins), and (3) methylation of the 16S ribosomal RNA amino-acyl site (acquired resistance). In particular, *A. baumannii* strains can acquire resistance genes at an alarming rate compared with other groups of bacteria. Chromosomal analysis of extensively drug-resistant *A. baumannii* strains has identified large resistance islands on the chromosome with up to 50 resistance genes (Imperi et al., 2011). These resistance islands are flanked by insertion sequences and encode transposase genes, indicating their recent origin from multiple genera of Gram-negative bacteria, including *P. aeruginosa, K. pneumoniae*, and *E. coli*. The acquisition of highly adaptable resistance determinants in the face of antibiotic-induced selective pressure necessitates the development of new therapies to treat infections caused by opportunistic bacterial pathogens.

Considering that no new approved antibacterial with a novel mechanism of action has been developed in the past 30 years, there has been an increase in the use of combination therapy with standard care drugs and the development of new drugs. There is a rapid rate of resistance to clinically important aminoglycosides (amikacin, gentamicin, tobramycin, and plazomicin) (Golkar et al., 2021; Bassenden et al., 2021). The occurrence of toxicity associated with aminoglycosides can narrow the window for their clinical use (Arya, 2007). However, in light of combination drug therapy, there is renewed interest. Combination therapy using tobramycin or amikacin and the last resort drug colistin is often necessary for treating invasive lung infections caused by Gram-negative bacteria harboring multidrug-resistant genes (Ramsey et al., 1999; Hodson et al., 2002; Fulnecky et al., 2005). Polymyxin B and colistin (polymyxin E) are both used in the clinical setting for treating bacterial infections, and it is recommended that colistin be administered in combination with one or more active antimicrobial agents, such as tobramycin. Polymyxins are composed of polycationic lipopeptides that interact with lipopolysaccharides of the Gram-negative bacterial outer membrane by binding divalent cations (Mg^2+^ and Ca^2+^), resulting in the loss of outer membrane integrity, eventually leading to cell lysis and death (Newton, 1956; Hancock, 1997; Evans et al., 1999; Hermsen et al., 2003). Aminoglycosides, such as tobramycin and neomycin, exert antibacterial activity by binding to the amino-acyl site (A-site) of the 16S rRNA component of the 23S ribosomal subunit, which results in the inhibition of protein synthesis and ultimately cell death (Davis, 1987; Tevyashova and Shapovalova, 2021). It is thought that the mechanism of action of the aminoglycoside/colistin combination is because of an accelerated influx of tobramycin caused by cell membrane damage and heightened permeabilization of the outer membrane (OM), which induces more rapid cell death than either alone (Hurdle et al., 2011; Zou et al., 2018). Generally, the interaction between aminoglycosides and polymyxins, as used clinically, has an additive effect that can lower the concentrations required for each drug, thereby reducing toxicity (Almutairi, 2022; Bayatinejad et al., 2023; Wang et al., 2022; Güzel and Gerçeker, 2008; Zhu et al., 2022).

Several studies have demonstrated that conjugation of aminoglycosides with small molecules improves target binding and/or biological activity (Aradi et al., 2020; Arya, 2005; Charles and Arya, 2005; Jiang et al., 2015; Jin et al., 2016; Watkins et al., 2017; Ghosh et al., 2018; Kellish et al., 2014; Ranjan and Arya, 2019; McFarland et al., 2024; Kumar et al., 2013; Ranjan et al., 2020; Story et al., 2019; Watkins et al., 2019; Chandrika et al., 2018; Ramos et al., 2017; Degtyareva et al., 2017; Ranjan and Arya, 2016; Nahar et al., 2015; Ranjan and Arya, 2013; Willis and Arya, 2009), allowing the action of two (Xue et al., 2002) or more (Willis and Arya, 2009, 2010) parts of the conjugate (for example, the aminoglycoside and amino acid, peptide, or aromatic unit attached) to function together and present a synergistic effect against the target binding site. Positively charged amino acids can form hydrogen bonds with unpaired RNA bases, have strong electrostatic interactions with the negatively charged RNA backbone, and interact with the outer membrane (OM) of Gram-negative bacteria. Basic amino acids, such as arginine and lysine, when conjugated to aminoglycosides, kanamycin or gentamicin, have been shown to have increased affinity and selectivity for RNA targets (Jiang et al., 2015; Litovchick et al., 1999; Lapidot et al., 2004; Tevyashova and Shapovalova, 2021; Jin et al., 2016). Additionally, more than one mechanism of action was found for lysine-neomycin conjugates, allowing increased penetration (membrane action) and inhibition of efflux (Bera et al., 2011). Another example of molecules with multiple modes of action is conjugates of membrane-acting compounds with tobramycin, allowing both membrane action and inhibition of protein synthesis (Dhondikubeer et al., 2012; Du et al., 2015; Rebizant, 2022; Gambato et al., 2023). In our previous studies, mono- and di-amino acid conjugates of neomycin or kanamycin were synthesized with the goal of overcoming the mechanisms of antibiotic resistance via enzymatic inactivation and drug efflux through improvement of the affinity and selectivity toward the bacterial 16S rRNA A-site. Several conjugates of the 215-member peptide-neomycin (P-NEO) library had higher affinity for A-site rRNA than neomycin (NEO). P-NEO conjugates that were more effective than NEO contained cysteine, tryptophan, lysine, and/or arginine residues. However, in aminoglycoside-resistant and/or intrinsically polymyxin-resistant bacteria, several tryptophan-NEO conjugates are ineffective (Jiang et al., 2015; Jin et al., 2016; Kukielski et al., 2018).

We hypothesized that the OM of Gram-negative bacteria had limited permeability to some P-NEO conjugates and that increasing the permeability of the OM with a sub-minimal inhibitory concentration (sub-MIC) of polymyxin B (PB) would act synergistically with the P-NEO conjugates and improve their bactericidal effect. In this study, the antibacterial activity of a P-NEO conjugate library (Jiang et al., 2015) in combination with PB was assayed against several strains of aminoglycoside-resistant *A. baumannii, K. pneumoniae*, and *P. aeruginosa*. These include extensive pandrug-resistant bacteria harboring 16S rRNA methyltransferases. A significant synergy between P-NEO and PB was observed across bacterial genera. However, an additive to an indifferent effect was observed with the parent compound, NEO, and other aminoglycosides. The MICs for the top P-NEO and PB combinations were below the maximum concentration that could be achieved in the serum and below the minimal inhibitory concentration of the parent compound NEO. This synergistic action improves the bactericidal effect of P-NEO to achieve an increased immunity against antibiotic resistance, such that the use of much lower drug concentrations can potentially reduce possible aminoglycoside- and polymyxin-based nephrotoxicities.

## 2 Materials and methods

### 2.1 Bacterial strains and test compounds

Genotypic and phenotypic antibiotic resistance profiles of the bacterial strains used in this study are given in Table 1. The majority of the bacterial strains used in this study were obtained from the Centers for Disease Control (CDC) and Prevention's Antimicrobial Isolate Resistance Bank. For each bacterial isolate, the complete antibiotic susceptibility profiles and antibiotic resistance genes present can be found on the CDC website: cdc.gov/ARIsolateBank/Panel/AllIsolate. Other bacterial isolates were received or purchased from BEI Resources, the Antimicrobial Resistance Leadership Group (ARLG), and the American Type Culture Collection (ATCC). Polymyxin B and aminoglycosides were purchased as sulfate salts from various vendors. Concentrated stocks of control aminoglycosides, PB, and P-NEO conjugates were prepared in water, aliquoted in cryovials, and stored at −80°C until use. P-NEO conjugates were synthesized in our laboratory, and their purity and other characteristics have been reported previously (Jiang et al., 2015). Bacterial stocks were stored at −80°C until use. Isolates were maintained on Luria-Bertani agar plates and routinely checked for purity and identity verification.

**Table 1:** Relevant genotypic and phenotypic profiles of aminoglycoside-resistant bacterial strains were chosen to screen an NEO-peptide library.

**Bacterial strain**	**Aminoglycoside resistance^a^ and drug efflux genes**	**MIC (**μ**M)**				
		**PB**	**NEO**	**AMK**	**GEN**	**TOB**
Abau19606 ATCC	*ant(3″)-IIc, **arm*** **A** *, MdfA/Cmr, TolC/OpmH*	2 (I)	8 (S)	16	16	8
AbauOIFC137 BEI	*aph(6)-Ic, aph(6)-Id, aph(3″)-I*	4 (R)	32 (R)	>64	>64	>64
Abau0313 CDC	*aac(3)-Ia, aadA1, ant(3″)-IIa, aph(3′)-Ia*	2 (I)	4 (S)	64	32	32
Abau0283 CDC	*aph(3′)-Ic, aph(3′)-VIa, **arm*** **A**	1 (S)	64 (R)	>64	64	32
Abau0273 CDC	*aac(3)-IIa, aph(3′)-Ic, strA, strB*	1 (S)	>64 (R)	64	32	16
Abau1256 ARLG	*aphA6, adeR, intI1, intI2, intI3*	4 (R)	32 (R)	>64	>64	32
Abau1297 ARLG	*aadB, adeR*	1 (S)	4 (S)	4	8	16
Abau1310 ARLG	*aacC1, aphA6, adeR*	4 (R)	64 (R)	>64	64	64
AbauBC5 BEI	*ant(3″)-Ia, aac(3)-I, TolC (OpmH), EmrAB-OMF*	2 (I)	32 (R)	32	64	32
Kpn0347 CDC	*aac(6′)-Ib, aph(3′)-Ia*	1 (S)	8 (S)	16	4	16
Kpn0120 CDC	*aac(6′)-33, aac(6′)-Ib, aadA2 aadB*	2 (I)	32 (R)	64	32	32
Kpn0558 CDC	*aac(3)-IId, aac(6′)-Ib-cr, aadA1, aadA2, **arm*** **A**	2 (I)	2 (S)	>64	>64	>64
Kpn0555 CDC	*aadA1, aph(3′)-Ia, **rmt*** **F**	2 (I)	64 (R)	>64	>64	64
Paer0236 CDC	*aadB, aph(3′)-IIb, mexA, mexE*	2 (I)	8 (S)	16	8	32
Paer0239 CDC	*aac(6′)-IIa, aadB, aph(3′)-Ic, strA, strB, mexA, mexE*	4 (R)	>64 (R)	>64	>64	>64
Paer0245 CDC	*aac(6′)-29B, aph(3′)-IIb, mexA, mexE*	1 (S)	32 (R)	>64	64	32
Paer0668 CDC	None identified	1 (S)	8 (S)	16	4	1
Paer27853 ATCC	ATCC reference strain	1 (S)	64 (R)	4	8	1

aac, aminoglycoside acetyltransferase; ant, aminoglycoside 3″-nucleotidyltransferase; (AadA family) aad, aminoglycoside adenylyltransferase; aph, aminoglycoside phosphotransferase; armA, rmtF, 16S rRNA methylases; Omp, truncated porin; int1, integrase; int2, integrase; int3, integrase; MdfA/Cmr, multidrug efflux pump broad spectrum, multidrug efflux system; EmrAB-OMF, membrane fusion component; EmrA ade, drug efflux system; TolC/OpmH, outer membrane channel; mexA, mexE, drug efflux system; NEO, neomycin; AMK, amikacin; TOB, tobramycin; PB, polymyxin B; ND, no data; R, resistance to polymyxins and/or neomycin; S, susceptibility to neomycin. Note that there are no established MIC breakpoints for neomycin; therefore, a strain was deemed resistant to neomycin if its MIC was ≥8 μM. MIC resistance breakpoints ug/mL (μM), CLSI updated 2023: For *A. baumannii*, AMK: 64 (82), GEN: 8 (14), TOB: 16 (11). For *K. pneumoniae*, AMK: 16 (20), GEN: 8 (14), TOB: 8 (5.5). For *P. aeruginosa*, AMK: 16 (20), GEN: no longer recommended, and TOB: 4 (3). For PB, MIC ug/mL (μM) ≤ 2 (1) susceptible, >2–4 (1–3) intermediate, and ≥4 (3) resistant. Susceptible (S), Intermediate (I), Resistant (R). Bacteria were obtained from several repositories, including BEI, Biodefense and Emerging Infections Repository; ARLG, Antibiotic Resistance Leadership Group; ATCC, American Type Culture Collection; CDC, Centers for Disease Control. For the Centers for Disease Control (CDC) and Prevention's Antimicrobial Resistance Isolate Bank, disclaimer: “The resistance mechanisms listed were identified by analysis of whole genome sequence using the ResFinder database (last updated June 2, 2016, and accessed on October 25, 2016). This analysis did not include mutations that may result in antibiotic resistance or resistance determinants added to the newer versions of the ResFinder database or other antimicrobial resistance gene databases. Sequence accession numbers have been provided so that users can analyze the data on their own if so desired.” ND, no data for aminoglycoside resistance determinants. ^a^In bold emphasizes genes for 16S rRNA methyltransferases *armA* and *rmtF* that confer resistance to all clinically-relevant aminoglycosides.

### 2.2 Minimal inhibitory concentration determination

MIC assays were conducted according to the Clinical Laboratory Standards Institute (CLSI) guidelines (document M100-M07). Briefly, the MICs of control aminoglycosides and P-NEO conjugates were determined in triplicate using the broth microdilution method in Mueller–Hinton II cation-adjusted (MHII-CA) medium. To determine the MICs, the concentration range of each drug (0.019–64 μM) was prepared by serial dilution in MHII-CA broth. To identify the sub-MIC of each test compound, the IC_50_ and IC_25_ were determined from the MICs. Fetal bovine serum (20%) was used to determine the effect of serum proteins on the MIC for the selected drug and drug combinations. Microtiter 96-well polystyrene plates containing 10 μL of 10X stock of each drug were inoculated with 90 μl of each bacterial strain (final cell concentration adjusted to ~5 × 10^5^ cells/mL) and incubated at 37°C for 16–20 h. MHII-CA broth with and without bacterial inoculum was used as the positive and negative controls, respectively. Optical density at 600 nm was measured using a plate reader. The MIC was defined as the lowest concentration that showed ≥90% growth inhibition in the MHII-CA medium. The percent growth inhibition was determined using the following equation:


$$\begin{array}{l} \%\;Growth\;Inhibition=100-100x\frac{A_{drug}-A_{background}}{A_{control}-A_{background}} \end{array}$$

### 2.3 Single-point concentration synergy screening

Bacterial strains were used in a high-throughput single-point concentration screening assay to determine the efficacy of the additive effect of the drug combination of the sub-MIC of PB (PB) and each P-NEO conjugate at 5 μM. The sub-MIC PB for each strain varied (0.25–1 μM) depending on the PB susceptibility of the bacterial strain. The single-point assay was performed as follows: a 10X solution (50 μM) of each P-NEO conjugate was prepared from 1 mM stock, and 10 uL of each compound was aliquoted into the wells of a 96-well styrene plate using an automated liquid handler. Bacteria were grown to the early exponential phase in tryptic soy broth and diluted 10,000-fold to a final cell concentration of ~ 5 × 10^5^ cells/mL in an appropriate volume of MH II broth containing sub-MIC PB. The bacterial suspension was manually dispensed in 90 μL aliquots to the wells of the P-NEO test plates and mixed immediately using a multichannel pipettor to reduce the localized action of the concentrated P-NEO solution at the bottom of the plate. Controls included sterile water (sterility control), bacterial suspension without drug (growth control), sub-MIC PB, 5 μM NEO, sub-MIC PB + NEO, and minimal inhibitory concentration of NEO or PB that killed ≥ 90% of the bacterial culture (positive controls). Plates were incubated for 24–48 h in a humidified chamber at 37°C and bacterial growth was monitored by light absorbance (OD_600nm_) using a plate reader. The results were averaged from triplicate samples, and the variation was calculated as the standard deviation from the mean.

### 2.4 Checkerboard titrations

The two-way dilution checkerboard assay was conducted in MHII-CA broth for each P-NEO conjugate that exhibited 70%−100% growth inhibition with sub-MIC PB in the single-point concentration synergy screen (SPCSS) described above. Six 2-fold dilutions for each test compound stock in MHII-CA were performed to obtain a 20X P-NEO conjugate with a concentration range from 31.25 μM to 100 μM and a concentration range of 0.625–40 μM for PB. The first drug component dilution (PB) was added to the top row of a 96-well polystyrene plate and diluted. The second drug dilution was added to the first column (P-NEO) and diluted to produce a 10X checkerboard plate. Then, ten microliters from each well of the stock checkerboard plate were transferred using an automated liquid handler to 96-well assay plates. A 90 μL volume of exponential-phase bacteria at a cell density of 5 x 10^5^ CFU mL^−1^ was added to each well of the assay plate, and the plates were incubated at 37°C in a humidified chamber for 18–24 h. After the incubation period, the absorbance at OD_600_ was measured using a plate reader. The MIC and MIC in combination with ≥90% growth inhibition were used to determine the fractional inhibitory concentration index (FICI), which is defined as follows:


$$\begin{array}{l} FICI=\frac{FIC\;PB\;combination}{MIC\;PB\;alone}+\frac{FIC\;neo\;conjugate\;combination}{MIC\;neo\;conjugate\;alone} \end{array}$$

where FIC is the fractional inhibitory concentration in combination and MIC is the minimal inhibitory concentration.

### 2.5 Time kill analysis

Time-kill assays were performed on the combinations found to be “synergistic” or “additive” using the checkerboard method described above. Time-kill analysis was performed according to CLSI document M26-A guidelines. For each test compound and test combination, three polypropylene 50 mL tubes containing 10 mL of MHII-CA broth containing the drug or drug combination were inoculated with a mid-log-phase aliquot of the test strain to a density of ~5 × 10^5^ CFU mL^−1^ in a final volume of 10 ml and incubated in a shaking incubator at 37°C in ambient air. Aliquots were removed at 0, 2, 4, 6, 8, and 24 h post-inoculation and serially diluted in sterile 0.85% sodium chloride solution to determine the culturable counts. At each time point and for each of the three replicate assay tubes, 100 μL aliquots were spread-plated on 80 mm diameter nutrient agar plates in duplicate trypticase soy agar plates using a spiral plater. Total culturable bacteria (LOG_10_ CFU mL^−1^) were determined after 24 h of incubation at 37°C.

Synergy was defined as a ≥ 2- LOG_10_ decrease in colony count at 6, 8, or 24 h with the antimicrobial combination compared to the most active single agent. Indifference was defined as a <2-LOG_10_ increase or decrease in colony count at 6, 8, or 24 h with the combination compared with the most active drug alone. Antagonism was defined as a ≥2-LOG_10_ increase in colony count at 6, 8, or 24 h with the combination compared with that of the most active drug alone. Three time-kill assay tubes were used: untreated growth control, sterile control (MHII-CA broth without drug or bacterial inoculum), sub-MIC PB alone or sub-MIC NEO alone, MIC for PB or NEO, and 0.5×, 1× , and 2× the MIC for the combination found in the checkerboard synergy assay.

### 2.6 Microtiter plate assay for biofilm quantification of *A. baumannii* strains

For biofilm inhibition, 100 μl of 1 × 10^5^ cells mL^−1^ in Mueller Hinton II cation-adjusted (MHII-CA) broth was added to the wells of a high-binding polystyrene microtiter plate, and 100 μL of test solution was added to the cell suspension and mixed well by pipetting using a multichannel pipettor. Test solutions were as follows: MHII-CA (growth control), 0.4 μM PB, 4 μM NEO & 0.4 μM PB or 4 μM P-NEO & 0.4 μM PB. Assay plates were incubated for 16 h at 37°C. After incubation, planktonic cells were gently aspirated from the biofilm layer, and the biofilm was washed twice with phosphate-buffered saline (PBS; pH 7.4). After incubating the assay plates, biofilms were fixed with methanol for 15 min at room temperature. Fixed biofilms were washed twice with PBS, and 200 μl of 0.2% crystal violet solution was added to each well. After 5 min, excess crystal violet was removed by aspiration, the cells were washed twice with PBS, and they were air-dried. Cell-bound crystal violet was dissolved in 33% acetic acid, and the biofilm mass was quantified by spectroscopy (OD_570)_ using a microplate reader (SPARK, Tecan).

Biofilm eradication: Biofilms were pre-formed onto 96-well flat-bottom polystyrene microtiter plates as follows: 100 μl of exponential phase cells in MHII-CA with an OD_600_ of 0.1 was inoculated into each well, and the plate was incubated for 16 h at 37°C to allow attachment of cells and biofilm formation. After incubation, the biofilm was washed as described above and treated with 100 μL of test solutions containing MHII-CA (growth control), 0.8 μM PB, 8 μM NEO, and 0.8 μM PB or 8 μM P-NEO & 0.8 μM PB. Biofilms were incubated for 16 h at 37°C. After incubation, the procedures for crystal violet staining of biofilms outlined in the previous paragraph were performed.

### 2.7 Resistance development

A 14-day resistance development assay was performed using neomycin-susceptible strains *A. baumannii* 19606, *K. pneumoniae* 0558, and *P. aeruginosa* 0668. To prepare for the drug challenge, bacteria were grown overnight in MHII non-cation-adjusted (MHII) broth. After overnight incubation, the cell densities were adjusted to 5 × 10^5^ CFU mL^−1^, and 90 μL was dispensed into the wells of a 96-well non-binding microtiter plate containing 10X solutions of the drug or a combination over the dilution range above and below the MICs and incubated at 37°C for 24 h. The cell suspension was quickly mixed with the drug solution using a multichannel pipettor to avoid the localized effects of the drugs. For subsequent challenge days, the wells at the subinhibitory MIC were sampled and diluted to achieve an OD600 reading of 0.08 and then diluted again by mixing 2 μL of the cell suspension with 5 mL MHII broth. After adjusting for cell density, 90 μL of the cell suspension was dispensed into drug challenge plates. Growth was monitored by measuring absorbance at OD600 over a period of 14 days. Purity checks were performed every 2 days to identify whether cross-contamination occurred by spread plating from wells with the most diluted drug exposure. The minimum bactericidal concentration was determined on day 14 using the spread plate method. When resistance development was observed, the stability of resistance was determined by taking the day 14 contents at the MIC and suspending them in 2 mL MHII, taking the contents at the sub-MIC, suspending them in 10 mL MHII, and incubating overnight. After incubation, 5 × 10^5^ CFU mL^−1^ was dispensed onto the drug challenge plate and incubated for 24 h.

### 2.8 Statistical analysis

The primary single-point synergy screen data were expressed as the mean and standard deviation of triplicate data. The paired two-tailed Student's *t*-test was used to determine the differences between the untreated and treated bacteria in the biofilm assays. Two-way ANOVA with repeated measures and *post-hoc* Kruskal–Wallis and Mann–Whitney tests were performed for the time-kill data. Data were analyzed using OriginPro software.

### 3 Results

#### 3.1 Extensive aminoglycoside resistance among clinical isolates of *A. baumannii, K. pneumoniae*, and *P. aeruginosa*

**Figure 1:**
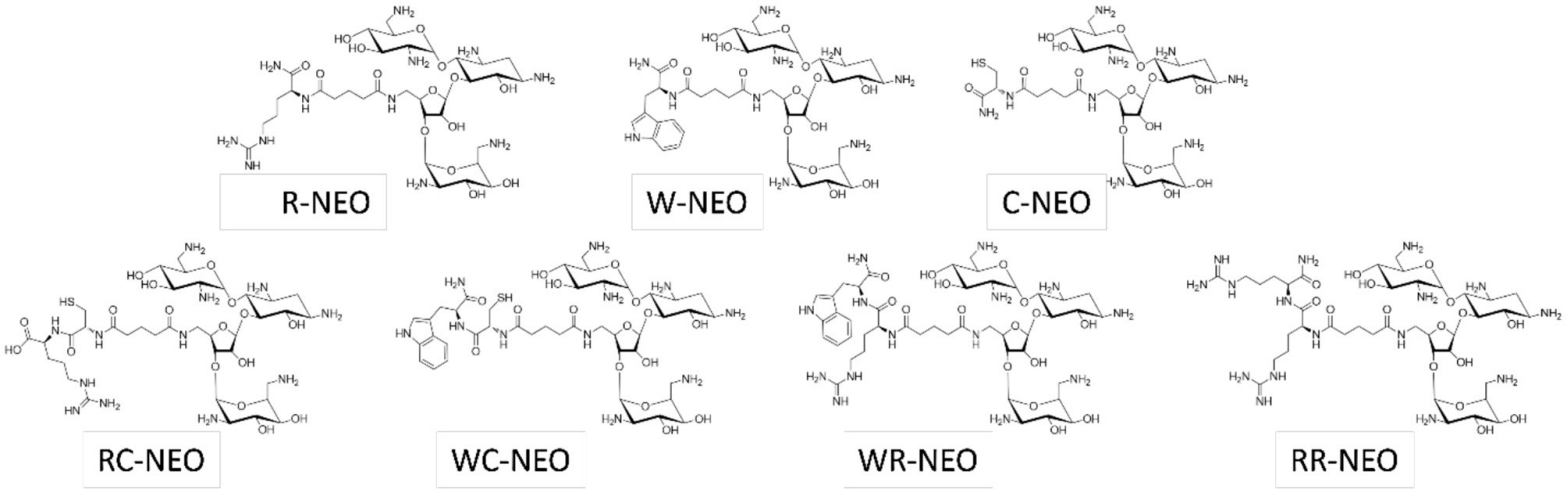
Structures of representative P-NEO conjugates.

**Figure 2:**
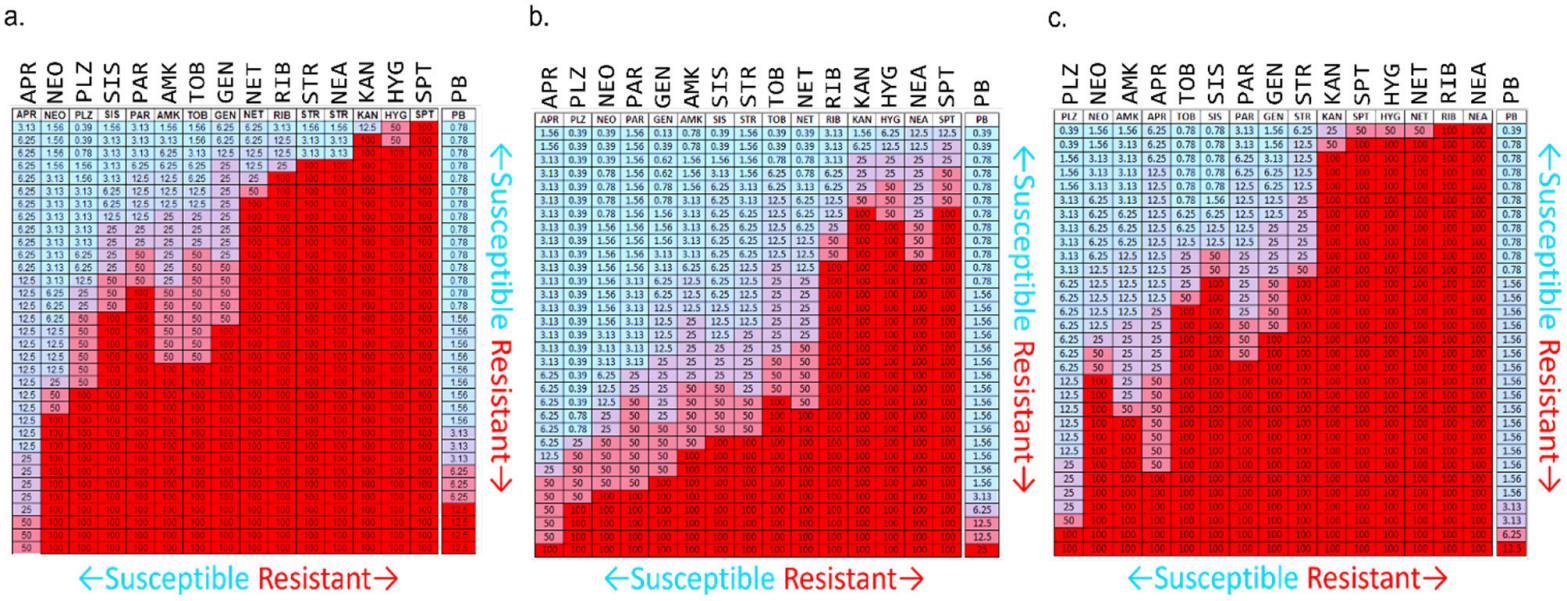
Susceptibility/resistance profiles for aminoglycosides and polymyxin B in ~30 clinical isolates of **(a)** *A. baumannii*, **(b)** *K. pneumoniae*, and **(c)** *P. aeruginosa*. Heat map interpretation: Blue to lavender = susceptible (MIC range 0.39–12.5 μM), purple to red = resistant (MIC range 25 to ≥100 μM). Numbers reflect the MIC for each aminoglycoside. The highest concentration used was 50 μM (purple cells); if an MIC was not found, 100 μM was administered instead (red cells). APR, apramycin; NEO, neomycin; PLZ, plazomicin; SIS, sisomicin; PAR, paromomycin; AMK, amikacin; TOB, tobramycin; GEN, gentamicin; NET, netilmicin; RIB, ribostamycin; STR, streptomycin; NEA, neamine; KAN, kanamycin; HYG, hygromycin; SPT, spectinomycin; PB, polymyxin B. Bacterial strains were acquired from the Centers for Infectious Disease Control and Prevention's Antimicrobial Resistance Bank, American Type Culture Collection, Antimicrobial Resistance Leadership Group, and BEI Resources. For a list of bacteria represented in this figure for aminoglycoside susceptibility profiling and their aminoglycoside resistance determinants (see Supplementary Table S2).

A list of P-NEO conjugates and their descriptions is provided in Supplementary Table S1 (refer to Figure 1 for representative P-NEO conjugates). Prior to the selection of bacteria for screening of the P-NEO library (Jiang et al., 2015), a panel of ~30 strains of clinical isolates of *A. baumannii, K. pneumoniae*, and *P. aeruginosa* was profiled for their susceptibility to a wide range of aminoglycosides, including NEO (Figures 2a–c). Clinical isolates were obtained from the Centers for Disease Control and Prevention's Antibiotic Resistance Isolate Bank, which has a large repository of bacteria collected from healthcare and food industries and communities across the United States. The aminoglycoside susceptibility profile differed between the three groups of bacteria with respect to NEO compared to plazomicin, the newest generation aminoglycoside. Many of these strains carried at least one gene encoding AME, and several strains carried three or more genes. Among the *A. baumannii* strains, 44% carried *arm*A, compared to *K. pneumoniae* (23%) and *P. aeruginosa* (6%), which carried either *arm*A or *rmt*A-G (Supplementary Table S2). Among the *A. baumannii* strains, approximately half were susceptible to NEO, and approximately one-third were susceptible to plazomicin (Figure 2a). For other aminoglycosides such as amikacin and tobramycin, *A. baumannii* susceptibility rates were low, with <30% susceptibility. *K. pneumoniae* strains showed greater susceptibility to most of the aminoglycosides tested (Figure 2b). The *P. aeruginosa* panel showed greater resistance to NEO but greater susceptibility to plazomicin (Figure 2c).

Based on the aminoglycoside susceptibility profiles shown in Figure 2, strains of *A. baumannii, K. pneumoniae*, and *P. aeruginosa* were selected for screening of the P-NEO library. Those that were chosen varied in susceptibility to NEO and PB and were mostly resistant to amikacin (AMK), gentamicin (GEN), and tobramycin (TOB) (Table 1, Supplementary Table S2). Selection was also based on the types of AME genes and 16S rRNA methyltransferases (Jouybari et al., 2021; Galimand et al., 2005, 2012; Yang and Hu, 2022) those carrying drug efflux genes and multidrug-resistant integrons (Vaillancourt et al., 2021; Richmond et al., 2016; Shetty et al., 2024), and susceptibility to PB. Both neomycin-resistant and sensitive strains are present in the selected panel, and 7 of the 18 strains were susceptible to NEO. Of the 18 strains, 7 were susceptible, 7 were intermediate, and 4 were resistant to PB. The majority of bacteria in the panel were also resistant to clinically relevant aminoglycosides, amikacin, gentamicin, and tobramycin.

#### 3.2 Primary screening of the P-NEO library reveals P-NEO conjugates with arginine, cysteine, tyrosine, and/or tryptophan are effective against aminoglycoside-resistant *A. baumannii*

**Figure 3:**
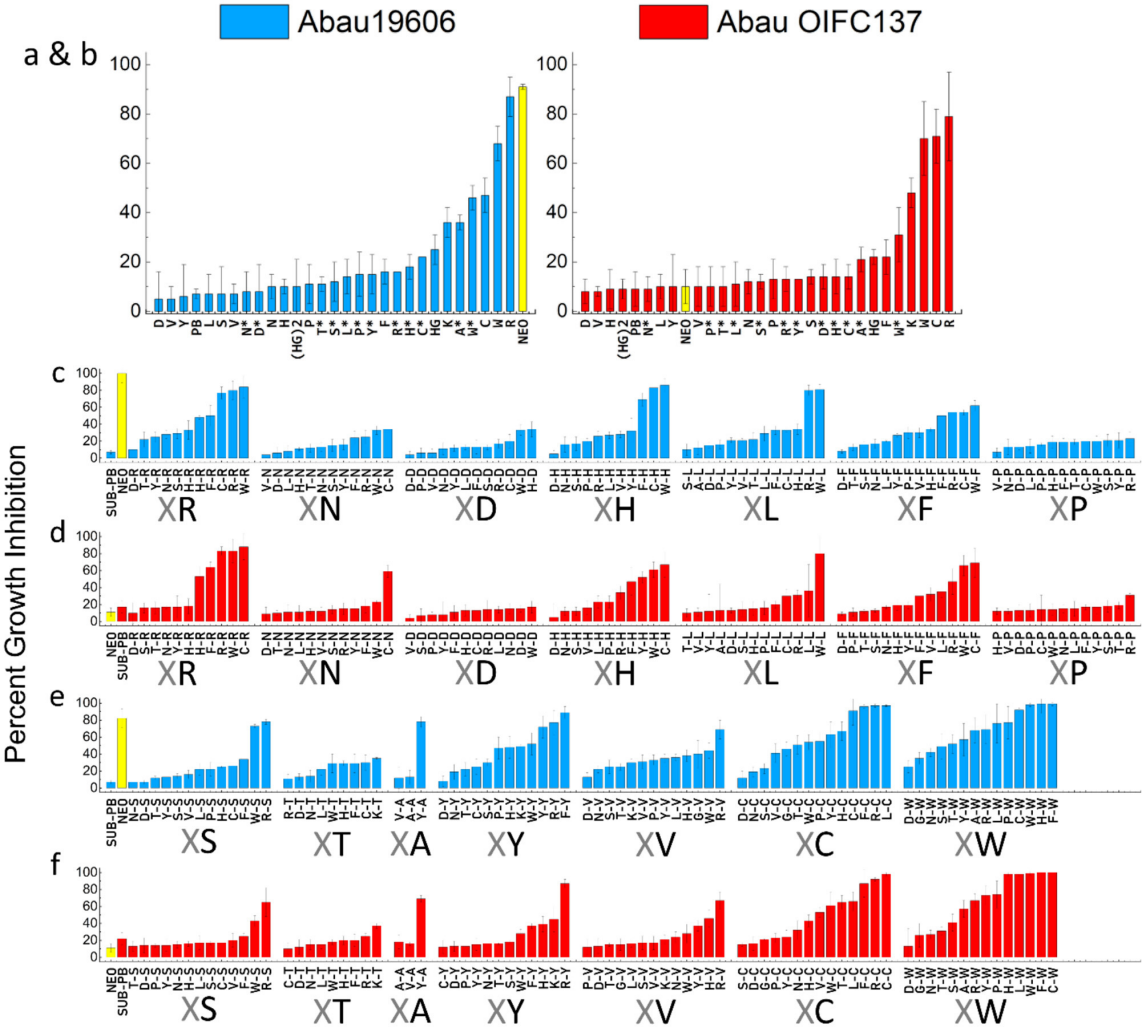
Percentage growth inhibition in a single-point synergy screen (SPCSS) of a P-NEO library (5 μM) in combination with sub-MIC of PB (PB) against *A. baumannii* strains. NEO-susceptible Abau19606 (blue = NEO^S^, MIC 4–8 μM), and NEO-resistant AbauOIFC137 (red = NEO^R^, MIC 32 μM). The sub-MIC of PB did not significantly affect growth after 24 h of incubation (≤ 20% growth inhibition). Neo linked with one amino acid **(a, b)** and with a β-alanine end group (marked with an asterisk*). NEO linked to two amino acids **(c–f)**, where **X** is any of the 18 amino acids, followed by the common amino acid linked to NEO (e.g., **X**-R-NEO, **X**-N-NEO and so on). For the common amino acid adjacent to the **X** amino acid and linked to NEO: R, Arginine; N, Asparagine; D, Aspartic acid; H, Histidine; L, Leucine; F, Phenylalanine; P, Proline; S, Serine; T, Threonine; A, Alanine; Y, Tyrosine; V, Valine; C, Cysteine; W, Tryptophan. Error bars represent the standard deviation of the mean of three replicates. The percent growth inhibition values are relative to growth control without PB, ≤ 20% growth inhibition is considered insignificant. Neomycin (NEO) was screened at 5 μM (yellow bar) and served as a control. Conjugates that inhibited growth by ≥70% in combination with PB were used in the checkerboard assays (refer to Supplementary Figures S2–S4 for an expanded presentation of this figure).

Previously, we observed little growth inhibition with P-NEO conjugates alone when screened against aminoglycoside-resistant bacteria, although several P-NEO conjugates demonstrated good binding affinity to 16S bacterial ribosomal A-site RNA using our fluorescent-neomycin binding displacement probe (Jiang et al., 2015; Watkins et al., 2013; Story and Arya, 2024; Watkins et al., 2015a; King et al., 2013). Therefore, a primary screen using a single-point concentration synergy screen (SPCSS) with 5 μM P-NEO and the specific sub-MIC PB for a particular strain was used to screen the P-NEO library. The P-NEO library is represented by conjugates of one or two L-form amino acids linked to NEO. The primary SPCSS began with NEO-susceptible (NEO^S^) and NEO-resistant strains (NEO^R^) of *A. baumannii* (Figure 3). Abau19606 had a NEO MIC of 8 μM, and AbauOIFC137 had a NEO MIC of 32 μM, respectively. These two *A. baumannii* strains also differed in their susceptibility to PB, with Abau19606 being susceptible and AbauOIFC137 being resistant. A comparison of the activity of the P-NEO conjugates between the NEO^S^ and NEO^R^ strains may indicate better target binding to the 16S rRNA A-site (Story and Arya, 2024), rather than better permeabilization facilitated by PB.

The primary SPCSS with P-NEO conjugates containing one L-form amino acid for NEO^S^ and NEO^R^ *A. baumannii* strains showed ≥70 growth inhibition for P-NEO conjugates with arginine (R), cysteine (C), and tryptophan (W) (Figures 3a, b). R^*^, C^*^, and W^*^ had an additional β-alanine end group, which was not as effective (20%−50% growth inhibition). Next, P-NEO conjugates with two L-form amino acids were compared (Figures 3c, d), where the end amino acid (**X**) is any of the 18 amino acids followed by a common amino acid that is linked to NEO (i.e., **X**R, **X**N, **X**D, and so on). For the diamino acid NEO group, we found that any combination of amino acids R, C, W, F, Y, S, and H was the most effective (≥ 80% growth inhibition) in NEO^S^ and NEO^R^ *A. baumannii* strains. Overall, NEO^R^ AbauOIF137 had a lower susceptibility to the P-NEO and PB combination than NEO^S^ Abau19606. The least effective P-NEO conjugates were those containing amino acids N, D, L, P, T, and A linked to NEO. Across Neo^S^ and NEO^R^ strains, the most effective P-NEO conjugates (with ≥80% growth inhibition) found in the SPCSS had a combination of R, W, H, F, and S.

#### 3.3 Checkerboard analysis of P-NEO conjugates supports a synergistic interaction between P-NEO conjugates and PB

**Figure 4:**
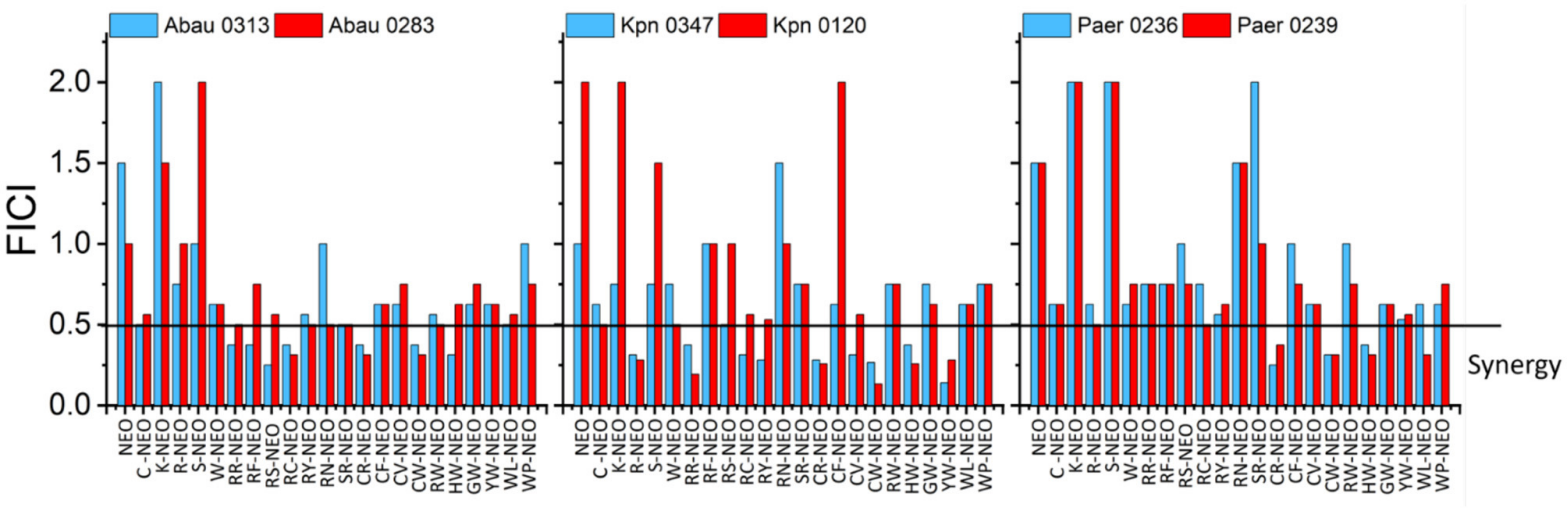
Comparison of fractional inhibitory concentration indices (FICI) derived from two-way checkerboard dilutions for the combination of the selected L-form amino acids P-NEO and PB in one NEO^S^ (blue) and one NEO^R^ (red) for *A. baumannii* (Abau0313, Abau0283), *K. pneumoniae* (Kpn0347, Kpn0120), and *P. aeruginosa* (Paer0236, Paer0239). FICI was calculated as follows: FICI = (FIC_P − NEO_/MIC_P − NEO_ + FIC_PB/_MIC_PB_). FICI interpretation: ≤ 0.5 indicates a synergistic interaction, >0.5–1.0 indicates an additive interaction, >1–4 indicates an indifferent interaction, and >4 indicates an antagonistic interaction. NEO, neomycin; C, cysteine; K, lysine; R, arginine; S, serine; W, tryptophan; F, phenylalanine; Y, tyrosine; N, asparagine; V, valine; H, histidine; G, glycine; P, proline. Because it was impractical to find the P-NEO MIC for some of the highly resistant strains, the FICI value was calculated using 64 μM as the cutoff. See Supplementary Table S3 for the MIC and FIC values from which FICI calculations were derived.

Although the SPCSS screen for the combination of P-NEO and PB against several NEO^S^ and NEO^R^ strains has provided some insight into which P-NEO conjugates may be most effective, SPCSS was susceptible to a high false-negative hit rate. Based on the primary screening, several P-NEO conjugates that included both negative and positive hits were selected for checkerboard analysis to determine the relationship between the interaction of P-NEO and PB (Figure 4). For this screening, the assay was extended to other bacteria with different aminoglycoside resistance mechanisms, including NEO^S^, NEO^R^, *A. baumannii, K. pneumoniae*, and *P. aeruginosa*. The checkerboard analysis included NEO as the control, and for all strains tested, an additive to indifferent effect was observed. The NEO MICs for the NEO^S^ strains Abau0313, Kpn0347, and Paer0236 were 4 μM, and those for the NEO^R^ strains Abau0283, Kpn0120, and Pear0239 were 64, 32, and 64 μM, respectively. MICs for P-NEO conjugates ranged from 16 to >64 μM across NEO^S^ and NEO^R^ strains, with NEO^S^ strains generally having lower MIC values (Supplementary Table S3). MICs for PB were in the intermediate range (1 μM) to the intermediate-resistant range (2–4 μM). For bacterial strains in which synergy was observed (Figure 4), the MIC in combination with P-NEO conjugates was reduced by 4–64-fold below its MIC (Supplementary Table S3). In contrast, the MIC for PB in combination was much narrower, being reduced by 2–4-fold. RC-NEO, CW-NEO, and HW-NEO demonstrated significant synergy with PB and *P. aeruginosa* strains, and the additive effects of the combination were primarily observed for other P-NEO conjugates.

#### 3.4 Time-kills demonstrate synergy for the RC-NEO and PB combination

**Figure 5:**
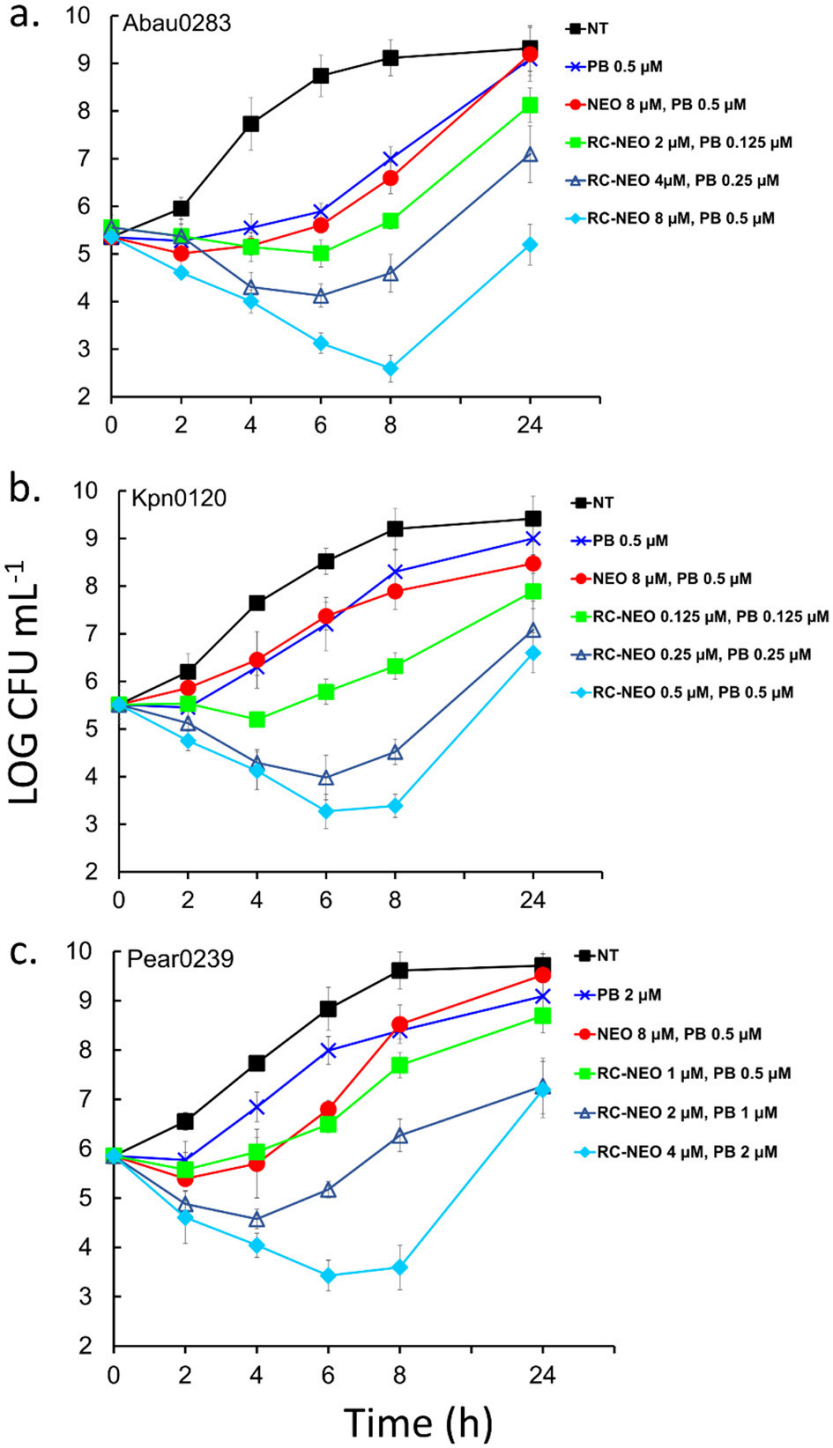
Time-kill curves of NEO-resistant strains of **(a)** extensively drug-resistant *A. baumannii* strain 0283, **(b)** extensively drug-resistant *K. pneumoniae* strain 0120, and **(c)** pandrug-resistant *P. aeruginosa* strain 0239 for RC-NEO and control NEO in combination with PB. LOG_10_ colony-forming units per milliliter (CFU mL^−1^) were monitored for 24 h. The concentrations used for the drugs alone and in combination reflect the concentrations used for each bacterial strain and were determined using the checkerboard synergy assay (Supplementary Table S3). Synergy was defined as a ≥2-LOG_10_ decrease in colony count at 6 or 8 h with the combination compared to the most active single agent (PB). Indifference was defined as a <2-LOG_10_ increase or decrease in colony count at 6, 8, or 24 h with the combination compared with the most active component. Antagonism was defined as a ≥2-LOG_10_ increase in colony count at 6, 8, or 24 h with the combination compared with that of the most active component alone. The NEO and PB MICs for *A. baumannii* (64 and 1 μM), *K. pneumoniae* (64 and 1 μM), and *P. aeruginosa* (>64 and 4 μM), respectively. Error bars represent the standard deviation from the mean of three replicate assay vials, with duplicate plating from each assay vial. Tests for significance: Two-way ANOVA with repeated measures was followed by *post-hoc* Bonferroni tests and additional non-parametric Kruskal–Wallis and Mann–Whitney tests for significance at *p* = 0.005 and *p* = 0.001. Data tables for the statistical metrics are presented in Supplementary Tables S5–S22.

For the time-kill assay, synergy was defined as a 2-log_10_ decrease in colony count by the combination compared with that by the most active single agent (PB), the most active combination (NEO+PB), or as a 2-log_10_ decrease in colony count compared with the starting inoculum. NEO^R^ strains of *A. baumannii* (Abau 0283, extensively drug-resistant), *K. pneumoniae* (Kpn 0120, extensively drug-resistant), and *P. aeruginosa* (Paer 0239, pandrug-resistant) were used. Colony-forming units per milliliter (CFU mL^−1^) were monitored for 24 h, and the following drug conditions were compared to the untreated control: sub-MIC for PB, sub-MIC for the combination of NEO+PB, and 0.5, 1, and 2 times the MICs found for the combination of RC-NEO and PB in the checkerboard analyses for each strain (Supplementary Table S3). Synergy was assessed in the 4–8 h range because of the resumption of growth by 24 h (Figure 5). For all strains, a bactericidal effect on PB was observed with a 3-fold reduction in the CFU mL^−1^ by 6 h and little to no recovery by 24 h, and NEO at MIC was bacteriostatic, with little change in the CFU/mL over 4 h and a gradual increase in growth by 24 h (data not shown). Some growth inhibition with sub-MIC of PB and NEO+PB was observed for most strains, with complete or near-complete recovery of growth after 24 h. A dose-dependent effect was observed for the combination of RC-NEO and PB at 0.5, 1, and 2 times the MIC. For *A. baumannii*, there was a significant difference between the combination of PB+NEO and PB+RC-NEO using twice the MIC at 4 h and 8 h, where a ≥2-fold LOG CFU mL^−1^ reduction with PB+RC-NEO was observed (*p* = 0.003). Additionally, a significant difference between 1 and 2 times the combination for RC-NEO+PB was found (*p* = 0.028) (Supplementary Tables S5–S10). Two-fold log reductions in CFU mL^−1^ for PB+RC-NEO compared to PB+NEO were found to be statistically significant for *K. pneumoniae* and *P. aeruginosa* using 1 and 2 times the RC-NEO+PB combination (Supplementary Tables S11–S22). Because of rebound growth after 8 h, a second administration of drugs between 6 and 8 h may extend the period of growth inhibition. The results from the time-kill assays support the SPCSS and checkerboard observations for the RC-NEO.

The effect of serum on the drug MIC and drug MIC in combination was evaluated in the neomycin-susceptible strains of *A. baumannii* ATCC 19606, *K. pneumoniae* CDC 0558, and *P. aeruginosa* CDC 0668 (Table 2). For PB and NEO, the MIC was generally unchanged, whereas the MICs for R-NEO, C-NEO, and RC-NEO increased by 2-fold. For the MICs in combination, no change was observed for *K. pneumoniae* and *P. aeruginosa*, whereas a 2–4-fold decrease was observed for the combination of PB and R-NEO, C-NEO, and RC-NEO with *A. baumannii*. Brightfield microscopy demonstrated changes in cell morphology with different treatments, where elongation of the cells and/or long chains of cells were observed with RC-NEO alone or in combination with PB, lysis of cells with PB, and very small cells with NEO (Supplementary Figure S6).

**Table 2:** Effect of serum on the minimal inhibitory concentration^a^ (MIC) of drugs alone and in combination with neomycin-susceptible bacteria.

	***A. baumannii**,* **ATCC 19606**		***K. pneumonia*** **, CDC 0558**		***P. aeruginosa*** **, CDC 0668**	
**Drug treatment**	**Without serum**	**20% serum**	**Without serum**	**20% serum**	**Without serum**	**20% serum**
PB	2–4	2	4	4	2	2
NEO	8	8–16	2	2–4	4–8	8
R-NEO	32	64	16	32	64	64
C-NEO	32	64	16	32	16	16
RC-NEO	32	64	32	32	16	32
NEO+PB	4/0.50	4/0.50	1/0.13	2/0.25	4/0.50	8/1
R-NEO+PB	1/0.13	0.5/0.06	1/0.13	0.5/0.06	1/0.13	4/0.50
C-NEO+PB	1/0.13	0.5 /0.06	2/0.25	1/0.13	2/0.25	4/0.50
RC-NEO+PB	1/0.13	0.25/0.03	1/0.13	1/0.13	2/0.25	2/0.25

^a^The MICs for the combinations are given for NEO, R-NEO, C-NEO, RC-NEO, and PB as NEO or P-NEO/PB.

#### 3.5 Inhibition of *A. baumannii* biofilm formation

**Figure 6:**
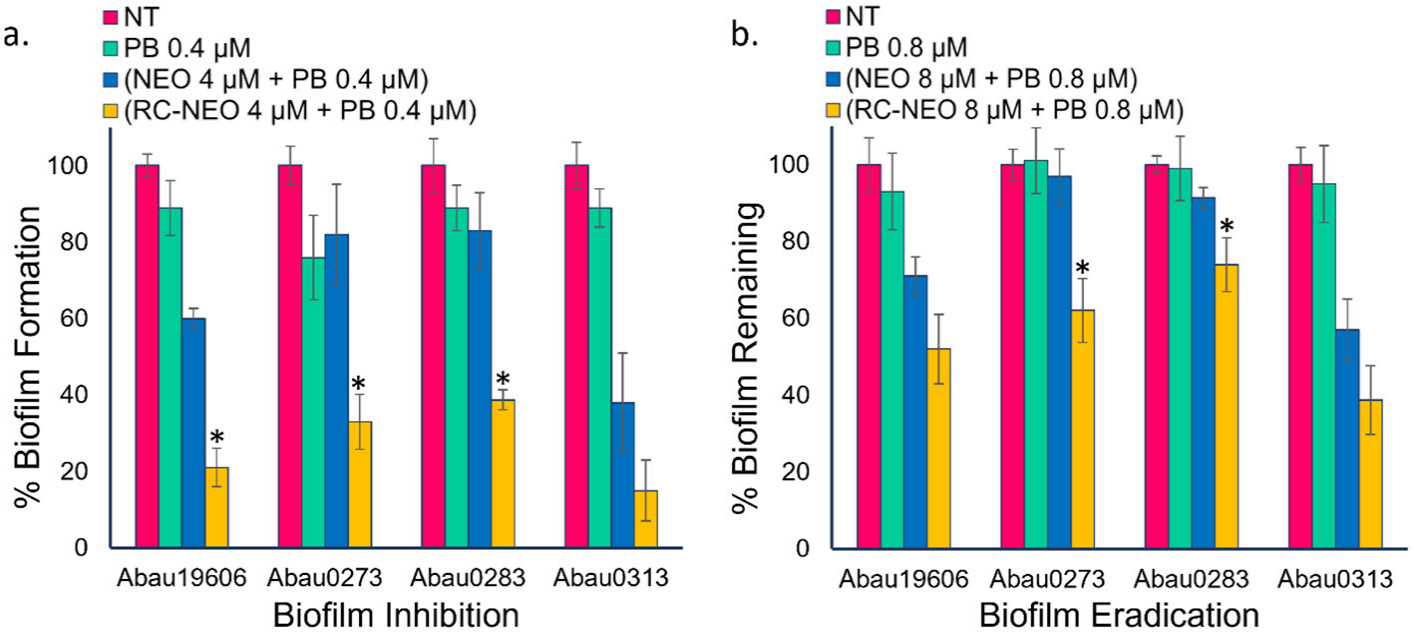
Biofilm inhibition and eradication indicate the percentage of biofilms formed or remaining in comparing four *A. baumannii* strains. **(a)** NT: no treatment growth control, PB: treatment with 0.4 μM PB alone, treatment with 4 μM NEO or RC-NEO in combination with 0.4 μM PB, **(b)** NT, no treatment growth control; PB, treatment with 0.8 μM PB alone, treatment with 8 μM NEO or RC-NEO in combination with 0.8 μM PB. Data are expressed as the mean ± standard deviation of triplicate data. The percent biofilm for the treatment groups was normalized to the biofilm formed or remaining for the untreated growth control, based on the crystal violet retained. Error bars represent the standard deviation of the mean of three replicates. A two-tailed Student's *t*-test was used to determine the differences in biofilm formation between the control (NEO+PB) and test P-NEO (RC-NEO+PB) combinations, where *p ≤ 0.001.

The time-kill curves demonstrated a synergistic interaction between P-NEO and PB. The time-kill assay specifically tested the effect of the drugs on cells in the planktonic state, which is much more susceptible to bactericidal and bacteriostatic actions of the drugs alone and in combination than in the biofilm-associated cell state. Using the same drug combinations as in the time-kill assay, biofilm formation and eradication were evaluated for four *A. baumannii* strains with different levels of NEO susceptibility and aminoglycoside resistance determinants (Figure 6). The biofilm formation for the RC-NEO & PB combination against *A. baumannii* strains relative to the untreated control was 21% for Abau19606, 37% for Abau0273, 39% for Abau0283, and 17% for Abau0313 (Figure 6a). The percentage of biofilm remaining for established *A. baumannii* biofilms by the combination of RC-NEO and PB for Abau19606, Abau0273, Abau0283, and Abau0313 was 52%, 63%, 77%, and 39% of the untreated growth control, respectively. Significant reductions in the RC-NEO & PB combination relative to the NEO & PB combination were observed for Abau19606, Abau0273, and Abau0283 (Figure 6b).

#### 3.6 Lack of resistance development to peptide-neomycin conjugates

**Figure 7:**
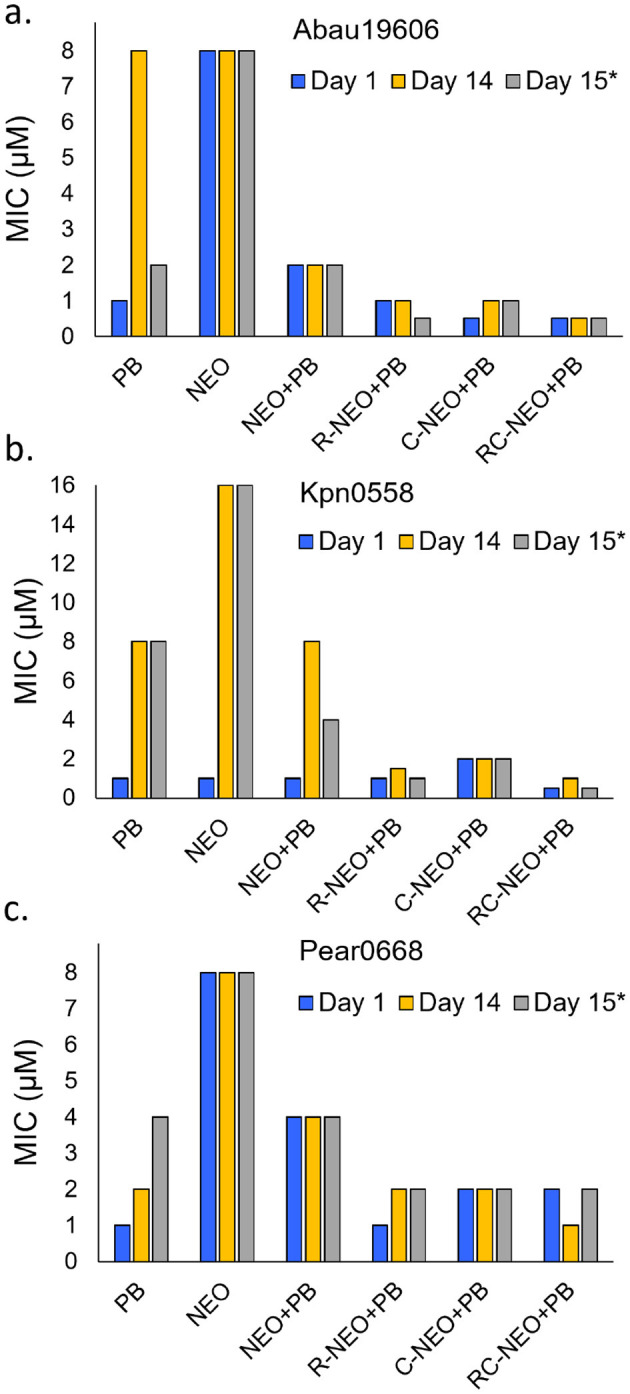
Test for resistance development comparing drug challenge on day 1 and day 14 as reflected in changes in the MIC for drugs alone or in combination. Day 15*: reflects the MICs after a 24 h removal of the drug or drug combination from day 14 cultures. **(a)** NEO susceptible *A. baumannii* 19606, **(b)** NEO susceptible *K. pneumoniae* 0558, **(c)** NEO susceptible *P. aeruginosa* 0668. The concentration range for PB administered alone was 0.125–8 μM in the combinations was 0.0625–4 μM. The MICs are given for NEO, R-NEO, C-NEO and RC-NEO in the combination with PB. The median MIC is presented for Day 14 where a trend in resistance was not observed over 14 days (Supplementary Tables S23–S25).

A 14-day resistance development assay was performed using the neomycin-susceptible strains *A. baumannii* 19606, *K. pneumoniae* 0558, and *P. aeruginosa* 0668 (Figure 7). Resistance development was not observed for the P-NEO conjugates R-NEO, C-NEO, and RC-NEO, alone or in combination with PB (Supplementary Tables S23–S25). In *A. baumannii* and *K. pneumoniae*, the MICs for the combinations demonstrated a narrow range of MICs for combinations of 1–2 μM, and for *P. aeruginosa*, the combination MIC range was 2–4 μM. By day 9, *A. baumannii* began to develop resistance to PB, with an 8-fold increase in MIC by day 14. However, this resistance was transient once the PB was removed (Figure 7). Resistance to NEO was not observed in *A. baumannii* and *P. aeruginosa* during this period. In contrast, *K. pneumoniae* developed significant resistance to both PB (8-fold increase in the MIC) and NEO (16-fold increase in the MIC) when administered alone, and this resistance appeared to be stable. Additionally, the MIC for the NEO+PB combination increased 4-fold in *K. pneumoniae*. It appeared that *P. aeruginosa* developed resistance to PB toward the end of the assay period, with a 4-fold increase in the MIC after the drug was removed for 24 h.

#### 3.7 Additive to indifferent effect for the combination of aminoglycosides and polymyxin B

The significant synergy observed between the P-NEO conjugates and PB was in stark contrast to that observed with NEO and PB, where an additive to indifferent relationship was found. To identify whether this lack of synergy with PB was unique to NEO or was a general rule for all aminoglycosides, ten other aminoglycosides in combination with PB were assessed with several strains of *A. baumannii* (Table 3). Generally, for strains exhibiting susceptibility, an additive effect was observed, and for those highly resistant, indifference to antagonism was found. One strain, *Acinetobacter radioresistens*, which is part of the normal flora of human skin, although susceptible to carbapenems and other antibiotics, is thought to be a source of carbapenem resistance and acquisition of other antibiotic resistance determinants in *A. baumannii* (Poirel et al., 2008; Lazarev et al., 2022). *A. radioresistens* is significantly more susceptible to aminoglycosides. A synergistic relationship with PB was observed with NEO, AMK, TOB, GEN, KAN, and PAR, for which the lowest MICs were observed. However, an additive to the indifferent relationship was observed for APR, SPT, STR, and RIB, where higher MICs were observed. Bacteria of other genera, such as *Klebsiella, Escherichia, Salmonella*, and *Shigella*, showed a similar pattern for the interaction of PB and the aminoglycosides amikacin and the latest-generation aminoglycoside plazomicin, which depended on the degree of susceptibility to the aminoglycoside (Supplementary Table S4). It is believed that both specific and indirect synergistic interactions occur. Notably, for highly susceptible strains, although the MIC for aminoglycosides in combination was markedly reduced (up to 16-fold), the reduction in the MIC for PB was at most 4-fold.

**Table 3:** Minimal inhibitory concentrations and fractional inhibitory concentration indices for the combination of aminoglycosides and PB in *A. baumannii* strains.

	**MIC**	**FICI**	**MIC**	**FICI**	**MIC**	**FICI**	**MIC**	**FICI**	**MIC**	**FICI**	**MIC**	**FICI**	**MIC**	**FICI**
**Drug**	**Abau19606**	**Abau19606**	**AbauBC5**	**AbauBC5**	**Abau1256**	**Abau1256**	**Abau1297**	**Abau1297**	**Abau1310**	**Abau1310**	**AbauOIFC137**	**AbauOIFC** **137**	**Arad** ^a^ **SK2**	**AradSK2**
NEO	3.13	0.75	50	2	50	2	3.13	1	50	1	50	2	0.78	0.39
AMK	12.5	2.5	25	2	100	>4	3.13	0.75	50	>4	100	1	0.78	0.39
TOB	3.13	0.75	6.25	0.75	50	2.25	25	2.25	50	>4	100	2	0.39	0.31
GEN	>100	2	50	1	>100	>4	>100	>4	>100	>4	>100	>4	1.56	0.5
KAN	12.5	1.25	25	2.25	>100	>4	100	>4	>100	>4	>100	>4	1.56	0.5
PLZ	50	1.5	3.13	1	25	1	1.56	0.5	50	2	50	2	0.19	0.39
PAR	25	1	50	1	>100	>4	3.13	1	>100	>4	>100	2	1.56	0.5
APR	12.5	1	25	2	12.5	2.5	12.5	1.5	12.5	0.63	25	1.5	3.13	0.75
SPT	>100	>4	>100	>4	>100	>4	>100	>4	>100	>4	>100	>4	12.5	2.25
STR	100	>4	>100	2	>100	>4	100	>4	>100	>4	>100	>4	6.25	0.75
RIB	50	1	>100	2	>100	>4	6.25	0.75	>100	>4	>100	>4	3.13	0.75
PB	1.56	NA	1.56	NA	3.13 (R)	NA	1.56	NA	3.13 (R)	NA	3.13 (R)	NA	0.78	NA

^*a*^Acinetobacter radioresistens.

MIC, minimal inhibitory concentration (μM); FICI, fractional inhibitory concentration index; NEO, neomycin; AMK, amikacin; TOB, tobramycin; GEN, gentamicin; KAN, kanamycin; PLZ, plazomicin; PAR, paromomycin; APR, apramycin; SPT, spectinomycin; STR, streptomycin; RIB, ribostamycin; PB, polymyxin B. FICI interpretation: ≤ 0.5 synergistic, >0.5–1 additive, >1–4 indifference, and >4 antagonistic. For instances where the MIC was not found, 100 μM was used to calculate the FICI, and at this concentration, bacterial growth was greater than that of the untreated control. R indicates resistance to polymyxin.

### 4 Discussion

We tested the growth inhibitory effect of a 191-member P-NEO library in combination with PB for extensively aminoglycoside-resistant and susceptible Gram-negative opportunistic pathogenic strains of *A. baumannii, K. pneumoniae*, and *P. aeruginosa*. The library was designed to compare the P-NEO conjugates with one or two amino acids. Each bacterial group in this study poses a unique challenge for drug treatment options because of differences in both acquired and intrinsic resistance factors. Previously, we identified members of the P-NEO library that did not serve as substrates for AMEs (Jiang et al., 2015). Besides AME genes, other acquired and intrinsic factors contribute to resistance to aminoglycosides. For example, there is a high prevalence of *A. baumannii* strains carrying *arm*A, an acquired resistance determinant that confers resistance to clinically relevant aminoglycosides. Intrinsic colistin heteroresistance is common among *A. baumannii* strains. *K. pneumoniae* produces a thick extracellular capsular material that is important for biofilm formation and supports intrinsic aminoglycoside tolerance and resistance. Consistently, clinical isolates of *K. pneumoniae* had a greater diversity of AME resistance determinants than either *A. baumannii* or *P. aeruginosa. P. aeruginosa* strains are especially well-equipped to upregulate their intrinsic drug efflux systems and employ quorum sensing in the establishment of biofilms and drug tolerance.

The combination of PB with specific P-NEO conjugates in aminoglycoside-resistant bacteria appeared not to serve as substrates for AMEs and bypass other resistance determinants as follows: (1) Previously, we found that P-NEO conjugates were not modified by purified AMEs (Jiang et al., 2015; Jin et al., 2016), and for the present study, all bacteria tested carried some variation of AME resistance determinants for aminoglycoside acetyltransferases and/or aminoglycoside phosphotransferases. (2) P-NEO conjugates are effective in bacteria carrying 16S rRNA methyltransferases such as *armA* and *rmt* genes, which included *A. baumannii* 0283 and *K. pneumoniae* strains 0558 and 0555. (3) The combination with PB allows uptake of P-NEO conjugates that alone cannot pass the outer membrane of Gram-negative bacteria and also allows uptake in capsulated bacteria (all *K. pneumoniae* tested). (4) It appears that drug efflux mechanisms are not sufficient for P-NEO export (all *P. aeruginosa* tested carried *mex*A and *mex*E drug efflux determinants). An additional factor that affects treatment options is the prevalence of colistin resistance among *A. baumannii*. In addition to the colistin resistance determinant *mcr*-1, *A. baumannii* can regulate the composition of the outer membrane lipid A or lose the lipid A portion through the expression of intrinsic factors, resulting in heteroresistance and complete resistance to colistin (Jean ShioShin and Hsueh PoRen, 2011; Moffatt et al., 2011; López-Rojas et al., 2011; Ko et al., 2007). However, the loss of the lipid A component of the outer membrane can increase *A. baumannii* susceptibility to drugs it is resistant to, including aminoglycosides (Moffatt et al., 2011, 2010; Mu et al., 2016; Carretero-Ledesma et al., 2018). Resistance did not develop with R-, C-, or RC-NEO conjugates alone or in combination with PB in the NEO-susceptible strains of *A. baumannii* 19606, *K. pneumoniae* 0558, and *P. aeruginosa* 0668. *A. baumannii* 19606 developed transient resistance to PB, *K. pneumoniae* 0558 developed a stable resistance to both PB and NEO, and *P. aeruginosa* 0668 developed resistance to PB toward the end of the drug-challenge time period. These bacteria host various acquired and intrinsic resistance factors. Resistance to polymyxins *in vitro* can develop via heteroresistance or chromosomal mutations, causing a transient or stable modification of lipid A and/or polysaccharides of the outer membrane (Andersson et al., 2019), and has been reported to occur rapidly among the populations of *A. baumannii, K. pneumoniae*, and *P. aeruginosa* strains *in vitro* (Thi Khanh Nhu et al., 2016; Janssen et al., 2020; Dößelmann et al., 2017). Surprisingly, the *K. pneumoniae* strain 0558 developed stable resistance to both PB and NEO. This strain has multiple AME genes and carries 16S rRNA methyltransferase *armA*. With the exception of NEO and apramycin, this strain was resistant to all clinically relevant aminoglycosides, including plazomicin (Supplementary Table S27 for the full aminoglycoside resistance profile and antibiotic resistance determinants).

P-NEO conjugates with cysteine (C), arginine (R), tryptophan (W), or tyrosine (Y) were the most effective, consistent with our previous study that analyzed the structure-activity relationship for P-NEO binding to the bacterial 16S rRNA A-site and bacterial growth inhibition. Previous studies (Jiang et al., 2015; Watkins et al., 2015b; Jin et al., 2016) demonstrated that the top P-NEO 16S rRNA A-site binders have a combination of W, Y, R, K, S, C, R, and H. The lowest MIC for *E. coli* was found with R-NEO (5 μM), followed by RN-NEO, RH-NEO, RS-NEO, RY-NEO, RV-NEO, RC-NEO, and RW-NEO (20 μM), well above the MIC for NEO (<1 μM). In contrast, the top P-NEOs for *P. aeruginosa* were WX-NEO, CX-NEO, XS-NEO, and XW-NEO (where X is any other amino acid). In the present study, the most effective P-NEO conjugates had the amino acid residues R, C, Y, and W, or combinations thereof. Notably, several strains carrying 16S rRNA methyltransferases were susceptible. Arginine carries a very stable positive charge among amino acids. The ionic interactions of arginine and PB with the outer membrane likely contributed to the synergistic relationship observed for *A. baumannii* and *K. pneumoniae* and the additive effects observed with *P. aeruginosa*. In bacteria, cysteine is the least abundant residue in structural proteins and is primarily found as a functional site in proteins (Marino and Gladyshev, 2012). Cysteine is cytotoxic to bacteria at low concentrations; thus, its intracellular concentration is highly regulated through its degradation and efflux as a part of sulfur assimilation (Takumi and Nonaka, 2016). Cysteine at low levels induces amino acid starvation, inhibits isoleucine synthesis, promotes reactive oxygen species, and elicits sulfide production, all contributing to cysteine toxicity (Sørensen and Pedersen, 1991; Korshunov et al., 2020; Marino and Gladyshev, 2012). It is unknown whether NEO conjugates with cysteine residues exhibit the same toxic properties as cysteine. C-NEO conjugates in combination with PB may result in the accumulation of C-NEO in the cytoplasm and may be resistant to intracellular degradation, in addition to the synergistic effects observed with PB. In addition to cysteine- and arginine-NEO conjugates, P-NEO with tryptophan and tyrosine residues appeared to be more effective against *P. aeruginosa* strains. Previous studies have demonstrated that both D- and L-isomers of tryptophan inhibit *P. aeruginosa* biofilm formation and disrupt quorum sensing (Brandenburg et al., 2013). The combination of amikacin and tyrosine inhibits *P. aeruginosa* biofilms (She et al., 2015). Another study found that the end modification of short antimicrobial peptides with one tryptophan residue enhanced the growth inhibitory activity of the peptide against *P. aeruginosa*, and additional tryptophan residues further improved the anti-pseudomonad activity. These tryptophan-enriched peptides were proposed to induce killing through cell wall and vesicle damage and increased binding to the LPS of the outer membrane (Pasupuleti et al., 2009). Our W-NEO conjugates, in combination with PB, may also inhibit biofilm formation in *P. aeruginosa* by binding to outer membrane LPS, in addition to binding to the RNA A-site.

For extensively drug-resistant Gram-negative bacteria, combination drug therapy is often deployed to treat infection. Colistin (polymyxin E) is considered a last-resort drug for the treatment of multidrug-resistant infections and is used in combination with amikacin or tobramycin, which have been shown to be effective in treating lung infections caused by multidrug-resistant bacteria (Tappenden et al., 2013; Herrmann et al., 2010; Taccetti et al., 2021; Buendía et al., 2024). Generally, the effect of the colistin/aminoglycoside combination has been shown to have an additive to indifferent relationship *in vitro* (Almutairi, 2022; Bayatinejad et al., 2023; Wang et al., 2022; Güzel and Gerçeker, 2008; Zhu et al., 2022). A synergistic relationship was found between P-NEO and PB, but at best, an additive effect was observed with NEO and other aminoglycosides for aminoglycoside-resistant bacteria and most of the susceptible bacteria. Based on the mechanism of action of aminoglycosides compared to PB, the data given in Table 3 and Supplementary Table S4 indicate both specific synergism and indirect synergism (Cokol et al., 2011). When the strain is highly aminoglycoside resistant because of aminoglycoside-modifying enzymes, drug efflux, and/or 16S rRNA methylases and polymyxin resistance factors, an antagonistic relationship prevails where the combination promotes growth, as observed in extensively drug-resistant *A. baumannii* strains (Ocampo et al., 2014). In highly aminoglycoside-susceptible strains, more extensive membrane damage results from both aminoglycoside and PB binding to the OM of Gram-negative cells. Initially, destabilization of the OM by PB would give aminoglycosides a “push” in passage across the cell wall and increased accumulation in the periplasmic space. Aminoglycoside entry into the cytoplasm is facilitated by the proton-motive force of the inner membrane. Once in the cell, irreversible binding to the target 16S rRNA A-site of the 30S ribosomal subunit induces misreading of the mRNA code and production of aberrant proteins. Misfolded truncated proteins cause further damage to the cell membrane, resulting in a surge of aminoglycosides in the cytoplasm, ultimately leading to cell death.

Bacterial biofilms pose a significant challenge in treating chronic infections, as biofilm cells are more tolerant to antibiotic treatment and grow rapidly on surfaces such as medical devices. The RC-NEO and PB combination was very effective in inhibiting biofilm growth but less effective in reducing pre-established *A. baumannii* biofilms, as it required four times more of the combination to observe a significant reduction.

## 5 Conclusions

In conclusion, we report a novel finding of a synergistic relationship between P-NEO and PB in both extensively drug- and pandrug-resistant bacteria, including those carrying 16S rRNA methyltransferase genes. In contrast, an additive to the indifferent relationship between aminoglycosides and PB was consistently observed in both aminoglycoside-sensitive and -resistant strains. P-NEO conjugates containing cysteine, arginine, or tryptophan residues were the most effective in synergy with PB, significantly lowering the MIC for P-NEO several-fold, with a smaller reduction in the MIC for PB. Amino acid-linked NEO can evade modification by AMEs and facilitate the “ESKAPE” of resistance development. Given that peptide modifications can be rapidly achieved using solution- or solid-phase chemistries, these findings provide promising new and rapidly tunable tools to mitigate the growth of drug-resistant pathogens using peptide-linked aminoglycosides.

## Data Availability

The original contributions presented in the study are included in the article/Supplementary material, further inquiries can be directed to the corresponding author.
