# Supplementary material for: Synergistic action between peptide-neomycin conjugates and polymyxin B against multidrug-resistant gram-negative pathogens
Source: Front Microbiol. 2025 Aug 7;16:1605813. doi: 10.3389/fmicb.2025.1605813 (PMC12367802; doi:10.3389/fmicb.2025.1605813)
Supplement: Supplementary file 1 [file Data_Sheet_1.pdf]

Synergistic Action Between Peptide-Neomycin Conjugates and Polymyxin B Against Multi-Drug-Resistant Gram-Negative Pathogens.

Sandra P. Story[2], Liuwei Jiang[1], Alain S. Leutou[2] and Dev P. Arya[1,2,*]
[1]*Laboratory of Medicinal Chemistry, Department of Chemistry, Clemson University, Clemson, South Carolina 29634, United States*
[2]*NUBAD, LLC, Greenville, South Carolina 29605, United States*

Content

**Table S1** List of P-NEO conjugates and description	Page S3
**Table S2** Aminoglycoside resistance genes for bacteria in the study.	Page S4
**Table S3** MIC, FIC and FICI values for L-form P-NEO and PB combinations.	Page S5-S6
**Table S4.** MIC and FIC values for the combination of aminoglycosides and PB in bacterial strains.	Page S7
**Table S5.** Two-way ANOVA w/ repeated measures for *A. baumannii* time-kill.	Page S8
**Table S6.** Bonferroini post-hoc test, treatment groups, *A. baumannii* time-kill.	Page S8
**Table S7.** Bonferroini post-hoc test, time (0 – 24 h), *A. baumannii* time-kill.	Page S9
**Table S8.** Kruskal-Wallis, treatment comparisons (0 – 24 h), *A. baumannii* time-kill.	Page S10
**Table S9.** Mann-Whitney, treatment comparisons (0 – 24 h), *A. baumannii* time-kill.	Page S11
**Table S10.** Mann-Whitney, treatment comparisons (6 – 8 h), *A. baumannii* time-kill.	Page S12
**Table S11.** Two-way ANOVA w/ repeated measures for *K. pneumoniae* time-kill.	Page S13
**Table S12.** Bonferroini post-hoc test, treatment groups, *K. pneumoniae* time-kill.	Page S13
**Table S13.** Bonferroini post-hoc test, time (0 – 24 h), *K. pneumoniae* time-kill.	Page S14
**Table S14.** Kruskal-Wallis, treatment comparisons (0 – 24 h), *K. pneumoniae* time-kill.	Page S15
**Table S15.** Mann-Whitney, treatment comparisons (0 – 24 h), *K. pneumoniae* time-kill.	Page S16
**Table S16.** Mann-Whitney, treatment comparisons (6 – 8 h), *K. pneumoniae* time-kill.	Page S17
**Table S17.** Two-way ANOVA w/ repeated measures for *P. aeruginosa* time-kill.	Page S18
**Table S18.** Bonferroini post-hoc test, treatment groups, *P. aeruginosa* time-kill.	Page S18
**Table S19.** Bonferroini post-hoc test, time (0 – 24 h), *P. aeruginosa* time-kill.	Page S19
**Table S20.** Kruskal-Wallis, treatment comparisons (0 – 24 h), *P. aeruginosa* time-kill.	Page S20
**Table S21.** Mann-Whitney, treatment comparisons (0 – 24 h), *P. aeruginosa* time-kill.	Page S21
**Table S22.** Mann-Whitney, treatment comparisons (6 – 8 h), *P. aeruginosa* time-kill.	Page S22
**Table S23 – Table S25.** MICs over a 14-day period for resistance development tests.	Page S23
**Table S26.** MBC with drugs alone and in combination at Day 1 and 14 of resistance development assay.	Page S24
**Table S27**. Antibiotic resistance profile and aminoglycoside resistance profile for *K. pneumoniae* 0558.	Page S24
**Figure S1 & Figure S2.** Additional SPSS bar graphs for the primary screen of the P-NEO library.	Page S25
**Figure S3 & Figure S4.** Additional SPSS bar graphs for the primary screen of the P-NEO library.	Page S26
**Figures S5.** Box plots of time-kills for *A. baumannii* 0283, *K. pneumoniae* 0120, *P. aeruginosa* 0239	Page S27
**Figure 6S.** Brightfield microscopy demonstrating changes in cell morphology with different treatments.	Page S28

Table S1. List of P-NEO conjugates and amino acid description.

**DPA L-Form A & AA-NEO (1 – 191)**[a]									
1	HG-Neo	43	HN-Neo	85	HL-Neo	127	RT-Neo	169	TC-Neo
2	W-Neo	44	TN-Neo	86	RL-Neo	128	DT-Neo	170	WC-Neo
3	N-Neo	45	NN-Neo	87	WL-Neo	129	NT-Neo	171	PC-Neo
4	D-Neo	46	SN-Neo	88	DF-Neo	130	LT-Neo	172	YC-Neo
5	H-Neo	47	YN-Neo	89	TF-Neo	131	WT-Neo	173	HC-Neo
6	L-Neo	48	FN-Neo	90	SF-Neo	132	HT-Neo	174	CC-Neo
7	F-Neo	49	RN-Neo	91	NF-Neo	133	FT-Neo	175	FC-Neo
8	P-Neo	50	WN-Neo	92	LF-Neo	134	CT-Neo	176	RC-Neo
9	S-Neo	51	CN-Neo	93	YF-Neo	135	KT-Neo	177	LC-Neo
10	V-Neo	52	DD-Neo	94	PF-Neo	136	AA-Neo	178	DW-Neo
11	Y-Neo	53	PD-Neo	95	VF-Neo	137	VA-Neo	179	GW-Neo
12	V-Neo	54	VD-Neo	96	HF-Neo	138	YA-Neo	180	NW-Neo
13	C-Neo	55	ND-Neo	97	FF-Neo	139	DY-Neo	181	SW-Neo
14	R-Neo	56	YD-Neo	98	RF-Neo	140	NY-Neo	182	TW-Neo
15	K-Neo	57	LD-Neo	99	CF-Neo	141	TY-Neo	183	YW-Neo
16	(HG)2-Neo	58	FD-Neo	100	WF-Neo	142	CY-Neo	184	AW-Neo
17	R*-Neo	59	SD-Neo	101	VP-Neo	143	SY-Neo	185	RW-Neo
18	N*-Neo	60	RD-Neo	102	NP-Neo	144	PY-Neo	186	LW-Neo
19	D*-Neo	61	CD-Neo	103	DP-Neo	145	HY-Neo	187	PW-Neo
20	H*-Neo	62	WD-Neo	104	LP-Neo	146	KY-Neo	188	CW-Neo
21	L*-Neo	63	HD-Neo	105	PP-Neo	147	WY-Neo	189	WW-Neo
22	P*-Neo	64	DH-Neo	106	HP-Neo	148	YY-Neo	190	HW-Neo
23	S*-Neo	65	NH-Neo	107	FP-Neo	149	RY-Neo	191	FW-Neo
24	T*-Neo	66	SH-Neo	108	TP-Neo	150	FY-Neo		
25	Y*-Neo	67	PH-Neo	109	CP-Neo	151	DV-Neo		
26	C*-Neo	68	RH-Neo	110	WP-Neo	152	NV-Neo		
27	W*-Neo	69	LH-Neo	111	SP-Neo	153	SV-Neo		
28	A*-Neo	70	VH-Neo	112	YP-Neo	154	TV-Neo		
29	DR-Neo	71	YH-Neo	113	RP-Neo	155	KV-Neo		
30	TR-Neo	72	FH-Neo	114	RS-Neo	156	VV-Neo		
31	YR-Neo	73	CH-Neo	115	NS-Neo	157	PV-Neo		
32	NR-Neo	74	WH-Neo	116	DS-Neo	158	YV-Neo		
33	SR-Neo	75	SL-Neo	117	TS-Neo	159	LV-Neo		
34	HR-Neo	76	AL-Neo	118	YS-Neo	160	HV-Neo		
35	HR-Neo	77	DL-Neo	119	SS-Neo	161	GV-Neo		
36	FR-Neo	78	PL-Neo	120	VS-Neo	162	WV-Neo		
37	CR-Neo	79	YL-Neo	121	LS-Neo	163	RV-Neo		
38	RR-Neo	80	VL-Neo	122	PS-Neo	164	DC-Neo		
39	WR-Neo	81	TL-Neo	123	HS-Neo	165	NC-Neo		
40	VN-Neo	82	LL-Neo	124	CS-Neo	166	SC-Neo		
41	DN-Neo	83	FL-Neo	125	FS-Neo	167	VC-Neo		
42	LN-Neo	84	CL-Neo	126	WS-Neo	168	GC-Neo		

[a]Synthesis and characterization has been described previously.[1]

Table S2. Aminoglycoside resistance genes for *A. baumannii*, *K. pneumoniae* and *P. aeruginosa*.

#	Strain	Aminoglycoside resistance genes	#	Strain	AME alleles
**1**	**Abau 19606**	***ant(3”)-IIc***	51	Kpn 0146	*strA, strB*
2	Abau 0312	*aph(3')-Ic*	52	Kpn 0659	*aph(3')-Ib, aph(6)-Id*
3	Abau 0301	*strA, strB*	53	Kpn 0362	*aac(6')-Ib, aph(3')-Ia*
4	Abau 0287	*strA, strB*	54	Kpn 0660	*aac(3)-IIa, aac(6')-Ib-cr*
5	Abau 0295	*strA, strB*	55	Kpn 0550	*aadA2, aadB*
6	Abau 0297	*strA, strB*	56	Kpn 0125	*aph(3')-Ia, aph(4)-Ia*
7	Abau 0296	*aadA2, aadB*	57	Kpn 0542	*aac(6')-Ib, aadA2*
**8**	**Abau 0313**	***aac(3)-Ia, aph(3')-Ic***	58	Kpn 0158	*aac(3)-IId, strA, strB*
9	Abau 0277	*aac(3)-IIa, strA, strB*	59	Kaer 0161	*aac(3)-IId strA, strB*
10	Abau 0284	*aac(3)-IIa, strA, strB*	60	Kpn 0361	*aac(6')-Ib, aph(3')-Ia, aph(4)-Ia*
11	Abau 0306	*aac(3)-IIa, strA, strB*	61	Kpn 0113	*aac(6')-Ib, aph(3')-Ia, aph(4)-Ia*
12	Abau 0289	*aac(3)-Ia, strA, strB*	62	Kpn 0135	*aac(3)-IIa, aac(6')-Ib, aph(3')-XV*
13	Abau 0292	*aac(3)-Ia, strA, strB*	63	Kpn 0138	*aadA2, strA, strB*
14	Abau 0293	*aac(3)-Ia, strA, strB*	64	Kpn 0553	*aac(3)-IId, aadA2, armA*
15	Abau 0304	*aac(3)-Ia, strA, strB*	65	Kpn 0658	*aadA2, aph(3')-Ia, aph(3')-Ib, aph(6)-Id*
16	Abau 0274	*aac(3)-Ia, aph(3')-Ic, strA, strB*	66	Kpn 0552	*aac(6')-Ib, aadA5, aph(3")-Ib, aph(6)-Id*
17	Abau 0280	*aac(3)-Ia, aph(3')-Ic, strA, strB*	**67**	**Kpn 0120**	***aac(6')-33, aac(6')-Ib, aadA2 aadB, aph(3')-Ia***
18	Abau 0294	*aac(3)-IIa, aph(3')-VIa, strA, strB*	68	Paer 0664	*aadA6*
19	Abau 0288	*armA, strA, strB*	69	Paer 0235	*aadB*
20	Abau 0308	*armA, strA, strB*	70	Paer 0513	*aadB*
**21**	**Abau 0273**	***aac(3)-IIa, aph(3')-Ic, aph(3')-VIa, strA, strB***	**71**	**Paer 0245**	***aac(6')-29B, aph(3')-IIb***
22	Abau 0311	*aph(3')-Ic, armA, strA, strB*	72	Paer 0528	*aph(3')-IIb*
23	Abau 0282	*aph(3')-Ic, armA, strA, strB*	73	Paer 0231	*aac(6')-IIc*
24	Abau 0299	*aph(3')-VIa, armA, strA, strB*	**74**	**Paer 0236**	***aadB, aph(3')-IIb***
25	Abau 0302	*aac(3)-Ia, armA, strA, strB*	75	Paer 0265	*ant(2")-Ia, aph(3')-IIb*
**26**	**Abau 0283**	***aph(3')-Ic, aph(3')-VIa, armA, strA, strB***	76	Paer 0357	*aadA6, aadB*
27	Abau 0278	*aph(3')-Ic, aph(3')-VIa, armA, strA, strB*	77	Paer 0355	*aph(6)-Id, strA*
28	Abau 0290	*aph(3')-Ic, aph(3')-VIa, armA, strA, strB*	78	Paer 0670	*aph(3')-Ib, aph(6)-Id*
29	Abau 0303	*aph(3')-Ic, aph(3')-VIa, armA, strA, strB*	79	Paer 0230	*aac(3)-Id, aadA2*
30	Abau 0299	*aph(3')-VIa, armA, strA, strB*	80	Paer 0242	*aac(3)-Id, aadA2*
31	Abau 0308	*armA, strA, strB,*	81	Paer 0248	*aac(3)-Id, aadA2*
32	Abau 0309	*aac(3)-Ia, armA, strA, strB*	82	Paer 0249	*aac(3)-Id, aadA2*
33	Abau 0310	*aac(3)-Ia, armA, strA, strB*	83	Paer 0241	*aac(6')-IIc, aadA7*
34	Abau 1274	*aac(6')-Ib-AKT, aadA1, ant(3")-IIa, aph(3')-Ia, aph(6)-Id, armA, strA*	84	Paer 0246	*aadB, rmtD2*
35	Kpn 0851	NR	85	Paer 0250	*aadB, rmtD2*
36	Kpn 0560	*aac(3)-IId, aac(6')-Ib-AKT, aadA1, armA*	86	Paer 0234	*aadA6, strA, strB*
37	Kpn 0557	*aac(6')-Ib-AKT, aadA2, aph(3')-VI, armA*	87	Paer 0669	*aadA2, aadA6, aadB*
**38**	**Kpn 0555**	***aac(6')-Ib-G, aadA1, aph(3')-Ia, rmtF1***	88	Paer 0353	*aac(6')-Il, aadA6, aph(3')-IIb*
**39**	**Kpn 0558**	***aac(3)-IId, aac(6')-Ib-cr, aadA1, aadA2, armA***	89	Paer 0666	*aadA6, aph(3')-VIb, aph(6)-Id, strA*
40	Kpn 0016	NR	90	Paer 0354	*aadA11, aadB, aph(6)-Id, strA*
41	Kpn 0657	NR	91	Paer 0515	*aac(3)-IIa, aadB, aph(3')-XV, strA*
42	Kpn 0012	*aac(3)-Iva, aac(6')-Ib, aadA1, aph(4)-Ia*	**92**	**Paer 0239**	***aac(6')-IIa, aadB, aph(3')-Ic, strA, strB***
43	Kpn 0856	*armA*	93	Paer 0508	*aadA2, aadB, aph(3')-VIb, aph(3')-XV, strA*
44	Kpn 0853	*aac(6')-IB3, rmtC*	**94**	**Paer 27853**	**NR**
45	Kpn 0145	*aadA1*	95	Paer 0232	*aadA6, strA, strB,*
46	Kpn 0152	*aadA1*	96	Paer 0235	*aadB*
47	Kpn 0117	*aadB*	97	Paer 0240	*aadA6*
48	Kpn 0548	*aph(3")-Ib aph(6)-Id*	98	Paer 0243	*aac(3)-Id, aadA2*
49	Koxy 0147	*aadB, aph(3')-Ic*	99	Paer 0255	*aac(3)-Id, aadA2*
**50**	**Kpn 0347**	***aac(6')-Ib, aph(3')-Ia***	100	Paer 0252	*aadA1, aadA6*

In bold are the strains chosen for screening the P-NEO library. Abbreviations: *aac*: aminoglycoside acetyltransferase; *ant*, *aad*: aminoglycoside adenylyltransferase; *aph*: aminoglycoside phosphotransferase; *armA, rmtF*: 16S rRNA methylases. NEO: neomycin; AMK: amikacin; TOB: tobramycin; PB: polymyxin B; NR: not reported. Bacteria were received from ATCC: American Type Culture Collection and the Centers for Disease Control. For Centers of Disease Control and Prevention’s Antimicrobial Resistance Isolate Bank, disclaimer: “The resistance mechanisms listed were identified by analysis of whole genome sequence using the ResFinder database (last updated June 2, 2016 and accessed on October 25, 2016). This analysis does not include mutations that may result in antibiotic resistance or resistance determinants added to newer versions of the ResFinder database or other antimicrobial resistance gene databases. Sequence accession numbers have been provided so that users can analyze the data on their own if so desired.” For complete antibiotic susceptibility profiles and list of all antibiotic resistance determinants refer to the CDC ARIsolate Bank website: cdc.gov/ARIsolateBank/Panel/AllIsolate.

Table S3. MIC, FIC (μM) and FICI calculated for the combination of L-form P-NEO and PB combinations.
(MIC for selected P-NEO run separate from checkerboard plate in bold)

Compound	**Kpn 0120**					**Kpn 0347**					**Kpn 0555**				
Drug	**FICI**	PB MIC	P-NEO MIC	P-NEO FIC	PB FIC	**FICI**	PB MIC	NEO MIC	P-NEO FIC	PB FIC	**FICI**	PB MIC	NEO MIC	P-NEO FIC	PB FIC
NEO	**2.00**	1	4	32	1	**1.00**	1	64	2	0.5	**2.00**	2	64	64	2
C	**0.50**	1	32 **(64)**	8	0.25	**0.63**	1	32	4	0.5	**0.50**	2	64	16	0.5
K	**2.00**	1	32	32	1	**0.75**	1	32	8	0.5	**2.00**	2	64	64	2
RN	**1.00**	1	32	16	0.5	**1.50**	1	16	16	0.5	**1.50**	2	32	32	1
SR	**0.75**	1	32	8	0.5	**0.75**	1	32	8	0.5	**0.75**	2	64	16	1
R	**0.28**	1	16 **(32)**	0.5	0.25	**0.31**	1	8	0.5	0.25	**0.25**	2	32	4	0.25
S	**1.50**	1	32	32	0.5	**0.75**	1	32	8	0.5	**2.00**	2	64	64	2
W	**0.50**	1	32 **(64)**	8	0.25	**0.75**	1	64	32	0.25	**0.19**	2	64	4	0.25
RR	**0.19**	1	16 **(32)**	1	0.13	**0.38**	1	8	1	0.25	**0.31**	2	16	4	0.125
RF	**1.00**	1	32	16	0.5	**1.00**	1	32	16	0.5	**1.00**	2	32	16	1
RS	**1.00**	1	32 **(64)**	16	0.5	**0.50**	1	16	4	0.25	**0.38**	2	16	2	0.5
RC	**0.56**	1	16	1	0.5	**0.31**	1	16	1	0.25	**0.31**	2	16	1	0.5
RW	**0.75**	1	32	8	0.5	**0.75**	1	16	4	0.5	**0.75**	2	64	32	0.5
HW	**0.26**	1	64	0.5	0.25	**0.38**	1	64	8	0.25	**0.31**	2	64	4	0.5
GW	**0.63**	1	32	4	0.5	**0.75**	1	64	16	0.5	**0.63**	2	64	8	1
RY	**0.53**	1	32	1	0.5	**0.28**	1	16	0.5	0.25	**0.27**	2	64	1	0.5
YW	**0.28**	1	64	2	0.25	**0.14**	1	32	0.5	0.125	**0.19**	2	64	4	0.25
CR	**0.26**	1	32 **(64)**	0.25	0.25	**0.28**	1	32	1	0.25	**0.28**	2	32	1	0.5
CF	**2.00**	1	32	32	1	**0.63**	1	64	8	0.5	**0.28**	2	64	2	0.5
CV	**0.56**	1	32	2	0.5	**0.31**	1	32	2	0.25	**0.56**	2	64	4	1
CW	**0.13**	1	64	0.5	0.125	**0.27**	1	64	1	0.25	**0.16**	2	64	2	0.25
WL	**0.63**	1	32	4	0.5	**0.63**	1	32	4	0.5	**0.38**	2	64	8	0.5
WP	**0.75**	1	32	8	0.5	**0.75**	1	64	16	0.5	**0.63**	2	64	8	1

Table S3. Continued

	**Paer 0236**					**Paer 0239**					**Paer 0245**				
Drug	**FICI**	PB MIC	NEO MIC	P-NEO FIC	PB FIC	**FICI**	PB MIC	NEO MIC	P-NEO FIC	PB FIC	**FICI**	PB MIC	NEO MIC	P-NEO FIC	PB FIC
NEO	**1.50**	2	4	2	2	**1.50**	4	64	64	2	**1.50**	1	64	64	0.5
C	**0.63**	2	32	4	1	**0.63**	4	64 **(64)**	8	2	**0.38**	1	32	4	0.25
K	**2.00**	2	64	64	2	**2.00**	4	64	64	4	**2.00**	1	64	64	1
RN	**1.50**	2	32	32	1	**1.50**	4	32	32	2	**1.00**	1	64	32	0.5
SR	**2.00**	2	32	32	2	**1.00**	4	32	16	2	**1.50**	1	32	32	0.5
R	**0.63**	2	64	8	1	**0.50**	4	32 **(64)**	8	1	**0.50**	1	32	8	0.25
S	**2.00**	2	64	64	2	**2.00**	4	64	64	4	**2.00**	1	64	64	1
W	**0.63**	2	64	8	1	**0.75**	4	64 **(64)**	16	2	**0.63**	1	64	8	0.5
RR	**0.75**	2	32	8	1	**0.75**	4	16 **(32)**	4	2	**0.50**	1	32	8	0.25
RF	**0.75**	2	64	16	1	**0.75**	4	64	16	2	**1.00**	1	64	32	0.5
RS	**1.00**	2	64	32	1	**0.75**	4	32 **(64)**	8	2	**1.00**	1	64	32	0.5
RC	**0.75**	2	32	16	0.5	**0.50**	4	32	8	1	**0.26**	1	64	4	0.2
RW	**1.00**	2	64	32	1	**0.75**	4	64	16	2	**0.75**	1	64	16	0.5
HW	**0.38**	2	64	8	0.5	**0.31**	4	64	4	1	**0.38**	1	64	8	0.25
GW	**0.63**	2	64	8	1	**0.63**	4	64	8	2	**0.75**	1	64	16	0.5
RY	**0.56**	2	64	4	1	**0.63**	4	32	4	2	**0.63**	1	64	8	0.5
YW	**0.53**	2	64	2	1	**0.56**	4	64	4	2	**0.75**	1	64	16	0.5
CR	**0.25**	2	32	4	0.25	**0.38**	4	16 **(32)**	2	1	**0.56**	1	64	4	0.5
CF	**1.00**	2	32	16	1	**0.75**	4	32	8	2	**1.00**	1	64	32	0.5
CV	**0.63**	2	64	8	1	**0.63**	4	64	8	2	**1.00**	1	64	32	0.5
CW	**0.31**	2	64	4	0.5	**0.31**	4	64	4	1	**0.63**	1	64	8	0.5
WL	**0.63**	2	64	8	1	**0.31**	4	64	4	1	**0.75**	1	64	16	0.5
WP	**0.63**	2	64	8	1	**0.75**	4	64	16	2	**1.00**	1	64	32	0.5

Table S3. Continued

	**Abau 0273**					**Abau 0283**					**Abau 0313**				
Drug	**FICI**	PB MIC	NEO MIC	P-NEO FIC	PB FIC	**FICI**	PB MIC	NEO MIC	P-NEO FIC	PB FIC	**FICI**	PB MIC	NEO MIC	P-NEO FIC	PB FIC
NEO	**1.50**	1	64	64	0.5	**1.00**	1	64	32	0.5	**1.50**	2	4	2	2

C	**0.50**	1	32	8	0.25	**0.56**	1	64 **(64)**	4	0.5	**0.50**	2	16	4	0.5
K	**0.75**	1	64	16	0.5	**1.50**	1	64	64	0.5	**2.00**	2	32	32	2
RN	**1.00**	1	32	16	0.5	**0.50**	1	32	8	0.25	**1.00**	2	32	16	1
SR	**0.50**	1	64	16	0.25	**0.50**	1	64	16	0.25	**0.50**	2	32	8	0.5
R	**0.50**	1	32	8	0.25	**1.00**	1	32 **(64)**	16	0.5	**0.75**	2	16	8	0.5
S	**2.00**	1	64	64	1	**2.00**	1	64	64	1	**1.00**	2	64	32	1
W	**0.75**	1	64	16	0.5	**0.63**	1	64 **(>64 72%)**	8	0.5	**0.63**	2	64	8	1
RR	**0.50**	1	32	8	0.25	**0.50**	1	64 **(32)**	16	0.25	**0.38**	2	16	2	0.5
RF	**0.63**	1	64	8	0.5	**0.75**	1	32	8	0.5	**0.38**	2	32	8	0.25
RS	**0.56**	1	64	4	0.5	**0.56**	1	64 **(>64 58%)**	4	0.5	**0.25**	2	32	4	0.25
RC	**0.53**	1	32	1	0.5	**0.31**	1	64	4	0.25	**0.38**	2	16	4	0.25
RW	**0.63**	1	64	8	0.5	**0.50**	1	32	8	0.25	**0.56**	2	32	2	1
HW	**0.52**	1	64	1	0.5	**0.63**	1	64	8	0.5	**0.31**	2	64	4	0.5
GW	**0.75**	1	64	16	0.5	**0.75**	1	64	16	0.5	**0.63**	2	64	8	1
RY	**0.38**	1	64	8	0.25	**0.50**	1	64	16	0.25	**0.56**	2	64	4	1
YW	**0.53**	1	64	2	0.5	**0.63**	1	64	8	0.5	**0.63**	2	64	8	1
CR	**0.28**	1	32	1	0.25	**0.31**	1	64 **(64)**	4	0.25	**0.38**	2	32	4	0.5
CF	**0.56**	1	64	4	0.5	**0.63**	1	64	8	0.5	**0.63**	2	32	4	1
CV	**0.56**	1	64	4	0.5	**0.75**	1	64	16	0.5	**0.63**	2	32	4	1
CW	**0.38**	1	64	8	0.25	**0.31**	1	64	4	0.25	**0.38**	2	32	4	0.5
WL	**0.56**	1	64	4	0.5	**0.56**	1	64	4	0.5	**0.50**	2	32	8	0.5
WP	**0.75**	1	64	16	0.5	**0.75**	1	64	16	0.5	**1.00**	2	64	32	1

Checkerboard data for *A. baumannii*, (Abau), *K. pneumoniae* (Kpn) and *P. aeruginosa* (Paer) strains. The FICI was calculated as follows: FICI = ($FIC_{P\text{-}NEO}/MIC_{P\text{-}NEO}$ + FIC_{PB}/MIC_{PB}). FICI interpretation: ≤ 0.5 indicates a synergistic interaction, > 0.5 – 1.0 indicates an additive interaction, >1 – 4 indicates an indifferent interaction, > 4 indicates an antagonistic interaction. Abbreviations: NEO: neomycin, C: cysteine, K: lysine, R: arginine, S: serine, W: tryptophan, F: phenylalanine, Y: tyrosine, N: asparagine, V: valine, H: histidine, G: glycine, P: proline. Because it was impractical to find the P-NEO MIC for some of the highly resistant strains, the FICI value was calculated using 64 μM as a cutoff.

Table S4. Minimal inhibitory concentrations and fractional inhibitory concentration indices for the combination of aminoglycosides and PB in bacterial strains.

Bacterial strain	**MIC AMK**	**MIC PB**	**FICI**	Bacterial strain	**MIC PLZ**	**MIC PB**	**FICI**
Kpn NR15410	1.56	2	0.5	Paer 0236	0.19	2	0.25
Ecloa 0132	1.56	2	0.5	Paer 27853	0.19	2	0.25
Eco 25922	1.56	1	0.5	Kpn 0146	0.39	1	0.5
Kpn 0117	25	1	0.75	Kpn 0158	0.39	1	0.5
Kpn 0126	6.25	1	0.75	Eco 25922	0.39	1	0.5
Kpn 0152	25	1	0.75	Kpn 0126	0.39	1	0.5
Kpn 0347	25	2	0.75	Kpn 0361	0.39	1	0.5
Abau 0301	12.5	1	0.75	Kpn NR15410	0.78	2	0.75
Kpn 0158	3.13	1	0.75	Kpn 0152	0.39	1	0.75
Eco 0114	50	1	0.75	Cfre 0116	0.78	1	0.75
Sfle 0421	12.5	1	0.75	Kpn 0145	0.78	1	0.75
Cfre 0116	3.13	1	0.75	Kpn 0347	1.56	2	0.75
Kpn 0145	25	1	1	Kpn 0120	0.39	2	0.75
Kpn 0660	6.25	2	1	Abau 0301	1.56	1	1.5
Paer 0236	25	2	1	Kpn 0557	50	1	1
Kpn 0120	25	2	1	Paer 0245	6.25	2	1
Kpn 0557	100	1	1.5	Kpn 0660	0.78	2	1
Kpn 0146	50	1	1.5	Eco 0114	6.25	1	1
Kpn 0361	50	1	1.5	SSef 0127	50	2	1
SSen 0127	50	2	1.5	Sflex 0421	3.13	1	1
Abau 0283	100	1	1.5	Ecloa 0132	0.19	2	1
Paer 27853	50	1	2	Kpn 0851	25	2	1.5
Paer 0245	100	1	2	Kpn 0117	0.39	1	1.5
Kpn 0125	50	4	2	Abau 0283	100	2	1.5
Kpn 0851	100	2	2	Paer 0239	100	4	1.5
Pmir 0159	6.25	100	2	Kpn 0125	25	4	2
Paer 0239	>100	4	2	Pmir 0159	0.19	100	2

Abbreviations: AMK: amikacin, PB: polymyxin B, FICI: fractional inhibitory concentration index, Kpn: *Klebsiella pneumoniae*, Ecloa: *Enterobacter cloacae*, Eco: *Escherichia coli*, Abau: *Acinetobacter baumannii*, Sfle: *Shigella flexneri*, Cfre: *Citrobacter freundii*, Paer: *Pseudomonas aeruginosa*, SSen: *Salmonella Senftenberg*, Pmi: *Proteus mirabilis*.

Table S5. *A. baumannii* 0283, Two-Way ANOVA with repeated measures (treatment groups and time)

	Sum of squares	df	Mean Squares	F	p	η^2	η^2_p
NT, PB, NEO+PB, 0.5x RC-NEO+PB, 1x RC-NEO+PB, 2x RC-NEO+PB	128.25	5	25.65	2877.01	<.001	0.42	1
Time	101.32	5	20.26	34.69	<.001	0.33	0.94
RM Factor x Time	69.26	25	2.77	310.75	<.001	0.23	0.99
Residuals (Between Subjects)	7.01	12	0.58				
Residuals (Within Subjects)	0.53	60	0.01				

Time-kill analysis test for significance for changes in CFU mL-1 as a function of time and treatment.

Table S6. *A. baumannii* 0283, Bonferroni Post-hoc-Tests RM Factor, Treatment groups

**Treatment Comparison**		Mean diff.	Std. Error	t	p	95% CI lower limit	95% CI upper limit
NT	PB	1.34	0.265	5.059	.001	0.78	1.9
NT	NEO+PB	1.55	0.305	5.076	.001	0.9	2.19
NT	0.5x RC-NEO+PB	1.88	0.355	5.301	.001	1.13	2.63
NT	1x RC-NEO+PB	2.53	0.449	5.632	<.001	1.58	3.47
NT	2x RC-NEO+PB	3.55	0.551	6.446	<.001	2.39	4.72
PB	NEO+PB	0.21	0.051	4.05	.013	0.1	0.31
PB	0.5x RC-NEO+PB	0.54	0.136	3.99	.014	0.26	0.83
PB	1x RC-NEO+PB	1.18	0.244	4.85	.002	0.67	1.7
PB	2x RC-NEO+PB	2.21	0.393	5.633	<.001	1.38	3.04
NEO+PB	0.5x RC-NEO+PB	0.34	0.136	2.484	.356	0.05	0.62
NEO+PB	1x RC-NEO+PB	0.98	0.239	4.098	.011	0.48	1.48
NEO+PB	2x RC-NEO+PB	2.01	0.39	5.145	.001	1.18	2.83
0.5x RC-NEO+PB	1x RC-NEO+PB	0.64	0.114	5.633	<.001	0.4	0.88
0.5x RC-NEO+PB	2x RC-NEO+PB	1.67	0.261	6.385	<.001	1.12	2.22
1x RC-NEO+PB	2x RC-NEO+PB	1.03	0.172	5.951	<.001	0.66	1.39

Table S7. *A. baumannii* 0283, Bonferroni Post-hoc-Tests, Time

	Mean diff.	Std. Error	t	p
0h-2h	0.16	0.25	0.62	1
0h-4h	0.1	0.25	0.41	1
0h-6h	0.01	0.25	0.02	1
0h-8h	-0.51	0.25	-2	1
0h-24h	-2.58	0.25	-10.13	<.001
2h-4h	-0.05	0.25	-0.21	1
2h-6h	-0.15	0.25	-0.6	1
2h-8h	-0.67	0.25	-2.62	.335
2h-24h	-2.74	0.25	-10.75	<.001
4h-6h	-0.1	0.25	-0.39	1
4h-8h	-0.61	0.25	-2.41	.496
4h-24h	-2.68	0.25	-10.54	<.001
6h-8h	-0.51	0.25	-2.02	.996
6h-24h	-2.59	0.25	-10.15	<.001
8h-24h	-2.07	0.25	-8.13	<.001

Table S8. *A. baumannii* 0283, Kruskal-Wallis, treatment comparisons

Pairwise Comparisons Kruskal-Wallis Test	Test Statistic	Std. Error	Std. Test Statistic	p	Adj. p
NT - PB	18	10.44	1.72	.085	1
NT - NEO+PB	25.83	10.44	2.47	.013	0.2
NT - 0.5x RC-NEO+PB	29.64	10.44	2.84	.005	0.068
NT - 1x RC-NEO+PB	46.06	10.44	4.41	<.001	<.001***
NT - 2x RC-NEO+PB	64.81	10.44	6.21	<.001	<.001***
PB - NEO+PB	7.83	10.44	0.75	.453	1
PB - 0.5x RC-NEO+PB	11.64	10.44	1.11	.265	1
PB - 1x RC-NEO+PB	28.06	10.44	2.69	.007	0.108
PB - 2x RC-NEO+PB	46.81	10.44	4.48	<.001	<.001***
NEO+PB - 0.5x RC-NEO+PB	3.81	10.44	0.36	.715	1
NEO+PB - 1x RC-NEO+PB	20.22	10.44	1.94	.053	0.791
NEO+PB - 2x RC-NEO+PB	38.97	10.44	3.73	<.001	.003**
0.5x RC-NEO+PB - 1x RC-NEO+PB	16.42	10.44	1.57	.116	1
0.5x RC-NEO+PB - 2x RC-NEO+PB	35.17	10.44	3.37	.001	.011*
1x RC-NEO+PB - 2x RC-NEO+PB	18.75	10.44	1.8	.072	1

Adj. p: Values adjusted with Dunn-Bonferroni correction. *Significant $*p < 0.05$, ** $p < 0.01$, ***$p < 0.001$

Table S9. *A. baumannii* 0283, Mann-Whitney, treatment comparisons, 0 – 24 h

Abau0283 0 – 24 h

Descriptive Statistics	N	Min	Q1	Med	Q3	Max	Descriptive Statistics	N	Min	Q1	Med	Q3	Max
NT	18	5.2	5.9	8.3	9.1	9.7	PB+NEO	18	4.7	5.2	5.5	6.7	9.9
PB	18	4.9	5.3	5.8	7.1	9.5	1x RCNEO+PB	18	3.9	4.3	5.0	5.7	7.5
**Ranks**	**N**	**Mean Rank**	**Sum Rank**				**Ranks**	**N**	**Mean Rank**	**Sum Rank**			
NT	18	23	408				PB+NEO	18	22	400			
PB	18	14	258				1x RCNEO+PB	18	15	266			
**Test Stat**	**U**	**Z**	**Prob>|U|**				**Test Stat**	**U**	**Z**	**Prob>|U|**			
	237	2.357	0.018					229	2.104	0.035			

Descriptive Statistics	N	Min	Q1	Med	Q3	Max	Descriptive Statistics	N	Min	Q1	Med	Q3	Max
NT	18	5.2	5.9	8.3	9.1	9.7	PB+NEO	18	4.7	5.2	5.5	6.7	9.9
PB+NEO	18	4.7	5.2	5.5	6.7	9.9	2x RCNEO+PB	18	2.3	3.1	4.4	5.2	5.7
**Ranks**	**N**	**Mean Rank**	**Sum Rank**				**Ranks**	**N**	**Mean Rank**	**Sum Rank**			
NT	18	23	419				PB+NEO	18	25	458			
PB+NEO	18	14	247				2x RCNEO+PB	18	12	208			
**Test Stat**	**U**	**Z**	**Prob>|U|**				**Test Stat**	**U**	**Z**	**Prob>|U|**			
	248	2.705	0.007					287	3.939	<0.0001			

Descriptive Statistics	N	Min	Q1	Med	Q3	Max	Descriptive Statistics	N	Min	Q1	Med	Q3	Max
PB	18	4.9	5.3	5.8	7.1	9.5	1x RCNEO+PB	18	3.9	4.3	5.0	5.7	7.5
PB+NEO	18	4.7	5.2	5.5	6.7	9.9	2x RCNEO+PB	18	2.3	3.1	4.4	5.2	5.7
**Ranks**	**N**	**Mean Rank**	**Sum Rank**				**Ranks**	**N**	**Mean Rank**	**Sum Rank**			
PB	18	20	365				1x RCNEO+PB	18	22	403			
PB+NEO	18	17	302				2x RCNEO+PB	18	15	263			
**Test Stat**	**U**	**Z**	**Prob>|U|**				**Test Stat**	**U**	**Z**	**Prob>|U|**			
	194	0.981	0.327					232	2.199	0.028			

Descriptive Statistics	N	Min	Q1	Med	Q3	Max	Descriptive Statistics	N	Min	Q1	Med	Q3	Max
PB+NEO	18	4.7	5.2	5.5	6.7	9.9	PB	18	4.9	5.3	5.8	7.1	9.5
0.5x RCNEO+PB	18	4.7	5.1	5.5	5.8	8.6	0.5x RCNEO+PB	18	4.7	5.1	5.5	5.8	8.6
**Ranks**	**N**	**Mean Rank**	**Sum Rank**				**Ranks**	**N**	**Mean Rank**	**Sum Rank**			
PB+NEO	18	19	345				PB	18	21	380			
0.5x RCNEO+PB	18	18	321				0.5x RCNEO+PB	18	16	286			
**Test Stat**	**U**	**Z**	**Prob>|U|**				**Test Stat**	**U**	**Z**	**Prob>|U|**			
	174	0.364	0.716					209	1.471	0.141			

Table S10. *A. baumannii* 0283, Mann-Whitney, treatment comparisons, 6 – 8 h

**Abau0283**		**6 – 8 h**				
**Descriptive Statistics**	**N**	**Min**	**Q1**	**Median**	**Q3**	**Max**
PB+NEO	6	5.4	5.6	6.0	6.7	6.9
1x RC-NEO+PB	6	3.9	4.1	4.3	4.7	5.0
**Ranks**	**N**	**Mean Rank**	**Sum Rank**			
PB+NEO	6	9.5	57			
1x RC-NEO+PB	6	3.5	21			
**Test Statistics**	**U**	**Z**	**Prob>|U|**			
	36	2.802	0.005			
**Descriptive Statistics**	**N**	**Min**	**Q1**	**Median**	**Q3**	**Max**
PB+NEO	6	5.4	5.6	6.0	6.7	6.9
2x RC-NEO+PB	6	2.3	2.4	2.8	3.2	3.2
**Ranks**	**N**	**Mean Rank**	**Sum Rank**			
PB+NEO	6	9.5	57			
2x RC-NEO+PB	6	3.5	21			
**Test Statistics**	**U**	**Z**	**Prob>|U|**			
	36	2.802	0.005			

Table S11. *K. pneumoniae* 0120, Two-Way ANOVA with repeated measures (treatment groups and time)

	Sum of squares	df	Mean Squares	F	p	η^2	η^2_p
NT, PB, NEO+PB, 0.5x RC-NEO+PB, 1x RC-NEO+PB, 2x RC-NEO+PB	131.51	5	26.3	1785.92	<.001	0.45	0.99
Time	89.65	5	17.93	23.74	<.001	0.31	0.91
RM Factor x Time	60.59	25	2.42	164.57	<.001	0.21	0.99
Residuals (Between Subjects)	9.06	12	0.76				
Residuals (Within Subjects)	0.88	60	0.01				

Table S12. *K. pneumoniae* 0120, Bonferroni Post-hoc-Tests RM Factor, Treatment groups

		Mean diff.	Std. Error	t	p	95% CI lower limit	95% CI upper limit
NT	PB	0.79	0.12	6.577	<.001	0.53	1.04
NT	NEO+PB	0.82	0.125	6.569	<.001	0.56	1.09
NT	0.5x RC-NEO+PB	1.71	0.263	6.506	<.001	1.15	2.26
NT	1x RC-NEO+PB	2.66	0.418	6.369	<.001	1.78	3.55
NT	2x RC-NEO+PB	3.14	0.492	6.387	<.001	2.1	4.18
PB	NEO+PB	0.03	0.094	0.366	1	-0.16	0.23
PB	0.5x RC-NEO+PB	0.92	0.182	5.062	.001	0.54	1.31
PB	1x RC-NEO+PB	1.88	0.335	5.598	.001	1.17	2.58
PB	2x RC-NEO+PB	2.35	0.413	5.693	<.001	1.48	3.23
NEO+PB	0.5x RC-NEO+PB	0.89	0.157	5.64	<.001	0.56	1.22
NEO+PB	1x RC-NEO+PB	1.84	0.311	5.932	<.001	1.19	2.5
NEO+PB	2x RC-NEO+PB	2.32	0.385	6.032	<.001	1.51	3.13
0.5x RC-NEO+PB	1x RC-NEO+PB	0.96	0.164	5.826	<.001	0.61	1.3
0.5x RC-NEO+PB	2x RC-NEO+PB	1.43	0.245	5.856	<.001	0.92	1.95
1x RC-NEO+PB	2x RC-NEO+PB	0.48	0.093	5.118	.001	0.28	0.67

Table S13. *K. pneumoniae* 0120, Bonferroni Post-hoc-Tests, Time

	Mean diff.	Std. Error	t	p
0h-2h	0.03	0.29	0.09	1
0h-4h	-0.16	0.29	-0.54	1
0h-6h	-0.51	0.29	-1.76	1
0h-8h	-1.09	0.29	-3.77	.04
0h-24h	-2.57	0.29	-8.86	<.001
2h-4h	-0.18	0.29	-0.63	1
2h-6h	-0.53	0.29	-1.85	1
2h-8h	-1.12	0.29	-3.86	.034
2h-24h	-2.59	0.29	-8.94	<.001
4h-6h	-0.35	0.29	-1.22	1
4h-8h	-0.94	0.29	-3.23	.108
4h-24h	-2.41	0.29	-8.32	<.001
6h-8h	-0.58	0.29	-2.01	1
6h-24h	-2.06	0.29	-7.1	<.001
8h-24h	-1.47	0.29	-5.08	.004

Table S14. *K. pneumoniae* 0120, Kruskal-Wallis, treatment comparisons

	Test Statistic	Std. Error	Std. Test Statistic	p	Adj. p
NT - PB	12.08	10.44	1.16	.247	1
NT - NEO+PB	11.42	10.44	1.09	.274	1
NT - 0.5x RC-NEO+PB	28.94	10.44	2.77	.006	.083
NT - 1x RC-NEO+PB	50.22	10.44	4.81	<.001	<.001
NT - 2x RC-NEO+PB	56.5	10.44	5.41	<.001	<.001
PB - NEO+PB	-0.67	10.44	-0.06	.949	1
PB - 0.5x RC-NEO+PB	16.86	10.44	1.62	.106	1
PB - 1x RC-NEO+PB	38.14	10.44	3.65	<.001	.004
PB - 2x RC-NEO+PB	44.42	10.44	4.26	<.001	<.001
NEO+PB - 0.5x RC-NEO+PB	17.53	10.44	1.68	.093	1
NEO+PB - 1x RC-NEO+PB	38.81	10.44	3.72	<.001	.003
NEO+PB - 2x RC-NEO+PB	45.08	10.44	4.32	<.001	<.001
0.5x RC-NEO+PB - 1x RC-NEO+PB	21.28	10.44	2.04	.042	.623
0.5x RC-NEO+PB - 2x RC-NEO+PB	27.56	10.44	2.64	.008	.124
1x RC-NEO+PB - 2x RC-NEO+PB	6.28	10.44	0.6	.548	1

Adj. p: Values adjusted with Bonferroni correction.

Table S15. *K. pneumoniae* 0120, Mann-Whitney, treatment comparisons, 0 – 24 h

**Kpn0120**		**0 – 24 h**											
**Desc Stat**	**N**	**Min**	**Q1**	**Med**	**Q3**	**Max**	**Desc Stat**	**N**	**Min**	**Q1**	**Med**	**Q3**	**Max**
NT	18	5.2	6.1	8.0	9.0	9.9	PB+NEO	18	5.3	5.8	7.1	7.9	9.0
PB	18	5.1	5.6	6.6	8.4	9.3	0.5x RCNEO+PB	18	5.1	5.3	5.8	6.4	8.3
**Ranks**	**N**	**Mean Rank**	**Sum Rank**				**Ranks**	**N**	**Mean Rank**	**Sum Rank**			
NT	18	21	382				PB+NEO	18	22	404			
PB	18	16	285				0.5x RCNEO+PB	18	15	262			
**Test Stat**	**U**	**Z**	**Prob>|U|**				**Test Stat**	**U**	**Z**	**Prob>|U|**			
	211	1.519	0.129					233	2.231	0.026			
**Desc Stat**	**N**	**Min**	**Q1**	**Med**	**Q3**	**Max**	**Desc Stat**	**N**	**Min**	**Q1**	**Med**	**Q3**	**Max**
NT	18	5.2	6.1	8.0	9.0	9.9	PB+NEO	18	5.3	5.8	7.1	7.9	9.0
PB+NEO	18	5.3	5.8	7.1	7.9	9.0	1x RCNEO+PB	18	3.5	4.3	4.7	5.6	7.7
**Ranks**	**N**	**Mean Rank**	**Sum Rank**				**Ranks**	**N**	**Mean Rank**	**Sum Rank**			
NT	18	21	387				PB+NEO	18	25	456			
PB+NEO	18	16	280				1x RCNEO+PB	18	12	211			
**Test Stat**	**U**	**Z**	**Prob>|U|**				**Test Stat**	**U**	**Z**	**Prob>|U|**			
	216	1.677	0.094					285	3.860	<0.0001			
**Desc Stat**	**N**	**Min**	**Q1**	**Med**	**Q3**	**Max**	**Desc Stat**	**N**	**Min**	**Q1**	**Med**	**Q3**	**Max**
PB	18	5.1	5.6	6.6	8.4	9.3	PB+NEO	18	5.3	5.8	7.1	7.9	9.0
PB+NEO	18	5.3	5.8	7.1	7.9	9.0	2x RCNEO+PB	18	2.9	3.6	4.5	5.6	7.0
**Ranks**	**N**	**Mean Rank**	**Sum Rank**				**Ranks**	**N**	**Mean Rank**	**Sum Rank**			
PB	18	18	333				PB+NEO	18	26	465			
PB+NEO	18	19	334				2x RCNEO+PB	18	11	202			
**Test Stat**	**U**	**Z**	**Prob>|U|**				**Test Stat**	**U**	**Z**	**Prob>|U|**			
	162	0.000	1.000					294	4.145	<0.0001			
**Desc Stat**	**N**	**Min**	**Q1**	**Med**	**Q3**	**Max**	**Desc Stat**	**N**	**Min**	**Q1**	**Med**	**Q3**	**Max**
PB	18	5.1	5.6	6.6	8.4	9.3	1x RCNEO+PB	18	3.5	4.3	4.7	5.6	7.7
0.5x RCNEO+PB	18	5.1	5.3	5.8	6.4	8.3	2x RCNEO+PB	18	2.9	3.6	4.5	5.6	7.0
**Ranks**	**N**	**Mean Rank**	**Sum Rank**				**Ranks**	**N**	**Mean Rank**	**Sum Rank**			
PB	18	22	398				1x RCNEO+PB	18	20.7	372.5			
0.5x RCNEO+PB	18	15	269				2x RCNEO+PB	18	16.3	293.5			
**Test Stat**	**U**	**Z**	**Prob>|U|**				**Test Stat**	**U**	**Z**	**Prob>|U|**			
	227	2.025	0.043					202	1.234	0.217			

Table S16. *K. pneumoniae* 0120, Mann-Whitney, treatment comparisons, 6 – 8 h

**Kpn120**		**6 – 8 h**				
**Descriptive Statistics**	**N**	**Min**	**Q1**	**Median**	**Q3**	**Max**
PB+NEO	6	7.1	7.3	7.6	8.0	8.3
1x RC-NEO+PB	6	3.5	3.9	4.3	4.6	4.8
**Ranks**	**N**	**Mean Rank**	**Sum Rank**			
PB+NEO	6	9.5	57			
1x RC-NEO+PB	6	3.5	21			
**Test Statistics**	**U**	**Z**	**Prob>|U|**			
	36	2.802	0.005			
**Descriptive Statistics**	**N**	**Min**	**Q1**	**Median**	**Q3**	**Max**
PB+NEO	6	7.1	7.3	7.6	8.0	8.3
2x RC-NEO+PB	6	2.9	3.1	3.3	3.6	3.6
**Ranks**	**N**	**Mean Rank**	**Sum Rank**			
PB+NEO	6	9.5	57			
2x RC-NEO+PB	6	3.5	21			
**Test Statistics**	**U**	**Z**	**Prob>|U|**			
	36	2.802	0.005			

Table S17. *P. aeruginosa* 0239, Two-Way ANOVA with repeated measures (treatment groups and time)

	Sum of squares	df	Mean Squares	F	p	η^2	η^2_p
NT, PB, NEO+PB, 0.5x RC-NEO+PB, 1x RC-NEO+PB, 2x RC-NEO+PB	124.63	5	24.93	1628.18	<.001	0.4	0.99
Time	125.65	5	25.13	40.55	<.001	0.4	0.94
RM Factor x Time	54.89	25	2.2	143.41	<.001	0.18	0.98
Residuals (Between Subjects)	7.44	12	0.62				
Residuals (Within Subjects)	0.92	60	0.02				

Table S18. *P. aeruginosa* 0239, Bonferroni Post-hoc-Tests RM Factor, Treatment groups

		Mean diff.	Std. Error	t	p	95% CI lower limit	95% CI upper limit
NT	PB	0.73	0.096	7.57	<.001	0.52	0.93
NT	NEO+PB	1.09	0.199	5.449	.001	0.67	1.51
NT	0.5x RC-NEO+PB	1.34	0.191	7.042	<.001	0.94	1.75
NT	1x RC-NEO+PB	2.38	0.304	7.828	<.001	1.74	3.02
NT	2x RC-NEO+PB	3.26	0.499	6.537	<.001	2.21	4.32
PB	NEO+PB	0.36	0.156	2.302	.514	0.03	0.69
PB	0.5x RC-NEO+PB	0.62	0.121	5.107	.001	0.36	0.87
PB	1x RC-NEO+PB	1.65	0.23	7.201	<.001	1.17	2.14
PB	2x RC-NEO+PB	2.54	0.421	6.024	<.001	1.65	3.43
NEO+PB	0.5x RC-NEO+PB	0.26	0.114	2.269	.548	0.02	0.5
NEO+PB	1x RC-NEO+PB	1.29	0.209	6.189	<.001	0.85	1.73
NEO+PB	2x RC-NEO+PB	2.18	0.399	5.462	.001	1.34	3.02
0.5x RC-NEO+PB	1x RC-NEO+PB	1.04	0.13	7.973	<.001	0.76	1.31
0.5x RC-NEO+PB	2x RC-NEO+PB	1.92	0.327	5.871	<.001	1.23	2.61
1x RC-NEO+PB	2x RC-NEO+PB	0.88	0.242	3.659	.029	0.37	1.39

Table S19. *P. aeruginosa* 0239, Bonferroni Post-hoc-Tests, Time

	Mean diff.	Std. Error	t	p
0h-2h	0.39	0.26	1.49	1
0h-4h	0.05	0.26	0.19	1
0h-6h	-0.6	0.26	-2.28	.625
0h-8h	-1.49	0.26	-5.69	.001
0h-24h	-2.73	0.26	-10.38	<.001
2h-4h	-0.34	0.26	-1.3	1
2h-6h	-0.99	0.26	-3.77	.04
2h-8h	-1.88	0.26	-7.18	<.001
2h-24h	-3.12	0.26	-11.88	<.001
4h-6h	-0.65	0.26	-2.48	.438
4h-8h	-1.54	0.26	-5.88	.001
4h-24h	-2.78	0.26	-10.58	<.001
6h-8h	-0.89	0.26	-3.41	.078
6h-24h	-2.13	0.26	-8.1	<.001
8h-24h	-1.23	0.26	-4.7	.008

Table S20. *P. aeruginosa* 0239, Kruskal-Wallis, treatment comparisons

	Test Statistic	Std. Error	Std. Test Statistic	p	Adj. p
NT - PB	11.47	10.44	1.1	.272	1
NT - NEO+PB	20.17	10.44	1.93	.053	.8
NT - 0.5x RC-NEO+PB	23.14	10.44	2.22	.027	.399
NT - 1x RC-NEO+PB	44.03	10.44	4.22	<.001	<.001
NT - 2x RC-NEO+PB	56.86	10.44	5.45	<.001	<.001
PB - NEO+PB	8.69	10.44	0.83	.405	1
PB - 0.5x RC-NEO+PB	11.67	10.44	1.12	.264	1
PB - 1x RC-NEO+PB	32.56	10.44	3.12	.002	.027
PB - 2x RC-NEO+PB	45.39	10.44	4.35	<.001	<.001
NEO+PB - 0.5x RC-NEO+PB	2.97	10.44	0.28	.776	1
NEO+PB - 1x RC-NEO+PB	23.86	10.44	2.29	.022	.334
NEO+PB - 2x RC-NEO+PB	36.69	10.44	3.52	<.001	.007
0.5x RC-NEO+PB - 1x RC-NEO+PB	20.89	10.44	2	.045	.68
0.5x RC-NEO+PB - 2x RC-NEO+PB	33.72	10.44	3.23	.001	.019
1x RC-NEO+PB - 2x RC-NEO+PB	12.83	10.44	1.23	.219	1

Adj. p: Values adjusted with Bonferroni correction.

Table S21. *P. aeruginosa* 0239, Mann-Whitney, treatment comparisons, 0 – 24 h

Paer0239 0 – 24 h

**Desc Stat**	**N**	**Min**	**Q1**	**Med**	**Q3**	**Max**
NT	18	5.7	6.5	8.1	9.4	10.3
PB	18	5.4	6.0	7.4	8.4	9.5
**Ranks**	**N**	**Mean Rank**	**Sum Rank**			
NT	18	21	383			
PB	18	16	284			
**Test Stat**	**U**	**Z**	**Prob>|U|**			
	212	1.551	0.121			
**Desc Stat**	**N**	**Min**	**Q1**	**Med**	**Q3**	**Max**
NT	18	5.7	6.5	8.1	9.4	10.3
PB+NEO	18	5.0	5.6	6.5	8.6	9.9
**Ranks**	**N**	**Mean Rank**	**Sum Rank**			
NT	18	22	398			
PB+NEO	18	15	269			
**Test Stat**	**U**	**Z**	**Prob>|U|**			
	227	2.025	0.043			
**Desc Stat**	**N**	**Min**	**Q1**	**Med**	**Q3**	**Max**
PB	18	5.4	6.0	7.4	8.4	9.5
PB+NEO	18	5.0	5.6	6.5	8.6	9.9
**Ranks**	**N**	**Mean Rank**	**Sum Rank**			
PB	18	20	361			
PB+NEO	18	17	306			
**Test Stat**	**U**	**Z**	**Prob>|U|**			
	190	0.854	0.393			
**Desc Stat**	**N**	**Min**	**Q1**	**Med**	**Q3**	**Max**
PB	18	5.4	6.0	7.4	8.4	9.5
0.5x RCNEO+PB	18	5.2	5.8	6.3	7.8	9.0
**Ranks**	**N**	**Mean Rank**	**Sum Rank**			
PB	18	21	379			
0.5x RCNEO+PB	18	16	288			
**Test Stat**	**U**	**Z**	**Prob>|U|**			
	207.5	1.424	0.154			

**Desc Stat**	**N**	**Min**	**Q1**	**Med**	**Q3**	**Max**
PB+NEO	18	5.0	5.6	6.5	8.6	9.9
0.5x RCNEO+PB	18	5.2	5.8	6.3	7.8	9.0
**Ranks**	**N**	**Mean Rank**	**Sum Rank**			
PB+NEO	18	19	341			
0.5x RCNEO+PB	18	18	326			
**Test Stat**	**U**	**Z**	**Prob>|U|**			
	170	0.222	0.825			
**Desc Stat**	**N**	**Min**	**Q1**	**Med**	**Q3**	**Max**
PB+NEO	18	5.0	5.6	6.5	8.6	9.9
1x RCNEO+PB	18	4.4	4.9	5.5	6.3	7.8
**Ranks**	**N**	**Mean Rank**	**Sum Rank**			
PB+NEO	18	23	414			
1x RCNEO+PB	18	14	253			
**Test Stat**	**U**	**Z**	**Prob>|U|**			
	243	2.53158	0.01136			
**Desc Stat**	**N**	**Min**	**Q1**	**Med**	**Q3**	**Max**
PB+NEO	18	5.0	5.6	6.5	8.6	9.9
2x RCNEO+PB	18	3.1	3.7	4.2	5.9	7.8
**Ranks**	**N**	**Mean Rank**	**Sum Rank**			
PB+NEO	18	25	442			
2x RCNEO+PB	18	12	225			
**Test Stat**	**U**	**Z**	**Prob>|U|**			
	271	3.418	0.001			
**Desc Stat**	**N**	**Min**	**Q1**	**Med**	**Q3**	**Max**
1x RCNEO+PB	18	4.4	4.9	5.5	6.3	7.8
2x RCNEO+PB	18	3.1	3.7	4.2	5.9	7.8
**Ranks**	**N**	**Mean Rank**	**Sum Rank**			
1x RCNEO+PB	18	23	407			
2x RCNEO+PB	18	14	260			
**Test Stat**	**U**	**Z**	**Prob>|U|**			
	236	2.310	0.021			

Table S22. *P. aeruginosa* 0239, Mann-Whitney, treatment comparisons, 6 – 8 h

**Paer0239**		**6 – 8 h**				
**Descriptive Statistics**	**N**	**Min**	**Q1**	**Median**	**Q3**	**Max**
PB+NEO	6	6.6	6.7	7.6	8.6	8.9
1x RC-NEO+PB	6	5.0	5.1	5.6	6.3	6.6
**Ranks**	**N**	**Mean Rank**	**Sum Rank**			
PB+NEO	6	9.5	57			
1x RC-NEO+PB	6	3.5	21			
**Test Statistics**	**U**	**Z**	**Prob>|U|**			
	36	2.802	0.005			
**Descriptive Statistics**	**N**	**Min**	**Q1**	**Median**	**Q3**	**Max**
PB+NEO	6	6.6	6.7	7.6	8.6	8.9
2x RC-NEO+PB	6	3.1	3.1	3.5	3.8	4.0
**Ranks**	**N**	**Mean Rank**	**Sum Rank**			
PB+NEO	6	9.5	57			
2x RC-NEO+PB	6	3.5	21			
**Test Statistics**	**U**	**Z**	**Prob>|U|**			
	36	2.802	0.005			

Table S23. Minimal inhibitory concentration (MIC, μM) over a 14-day serial passage of *A. baumannii* 19606 into drugs and drug combinations.

A. baumannii ATCC 19606	Day													
Treatment	**1**	**2**	**3**	**4**	**5**	**6**	**7**	**8**	**9**	**10**	**11**	**12**	**13**	**14**	**15[b]**
PB[a]	1	1	2	1	2	2	2	2	4	4	8	8	4	8	2
NEO	8	4	8	8	4	8	8	4	8	8	8	8	8	8	8
R-NEO	32	32	64	16	16	16	32	32	16	16	16	32	16	16	16
C-NEO	32	64	64	32	32	32	32	64	32	32	32	32	32	32	32
RC-NEO	64	64	64	32	64	32	32	64	32	32	32	32	64	32	32
NEO+PB	2	2	2	2	1	1	2	1	2	2	2	2	4	2	2
R-NEO+PB	1	1	1	1	1	1	1	0.5	1	1	2	1	1	1	0.5
C-NEO+PB	0.5	1	1	1	1	1	0.5	0.5	1	1	1	1	0.5	2	1
RC-NEO+PB	0.5	1	0.5	0.5	0.5	0.5	1	0.5	0.25	0.25	0.25	0.5	0.5	0.5	0.5

Table S24. Minimal inhibitory concentration (MIC, μM) over a 14-day serial passage of *K. pneumoniae* 0558 into drugs and drug combinations.

K. pneumoniae CDC 0558	Day														
Treatment	**1**	**2**	**3**	**4**	**5**	**6**	**7**	**8**	**9**	**10**	**11**	**12**	**13**	**14**	**15[b]**
PB[a]	1	0.5	1	2	4	8	8	>8	>8	8	>8	8	8	>8	8
NEO	1	2	2	2	1	1	2	4	2	4	8	8	8	16	16
R-NEO	16	16	8	8	16	8	8	8	16	8	16	16	16	16	8
C-NEO	16	32	16	16	16	16	8	16	16	16	16	16	8	8	8
RC-NEO	32	32	32	16	32	16	32	32	32	32	16	32	16	16	16
NEO+PB	1	1	2	2	1	1	1	2	2	2	4	4	8	8	4
R-NEO+PB	1	2	2	1	0.5	0.5	1	2	1	2	2	2	1	2	1
C-NEO+PB	2	2	2	1	2	4	1	2	1	2	2	1	2	2	2
RC-NEO+PB	0.5	1	1	2	0.5	1	1	0.5	1	0.5	1	1	1	0.5	0.5

Table S25. Minimal inhibitory concentration (MIC, μM) over a 14-day serial passage of *P. aeruginosa* 0668 into drugs and drug combinations.

P. aeruginosa CDC 0668	Day														
Treatment	**1**	**2**	**3**	**4**	**5**	**6**	**7**	**8**	**9**	**10**	**11**	**12**	**13**	**14**	**15[b]**
PB[a]	1	1	2	1	1	2	2	2	4	2	4	4	2	4	4
NEO	8	8	16	8	8	8	8	8	16	8	16	8	8	16	8
R-NEO	32	32	64	64	32	32	32	64	32	32	32	32	32	32	32
C-NEO	16	16	8	16	16	16	16	8	16	8	16	8	8	8	8
RC-NEO	16	16	16	16	8	8	16	8	8	16	8	16	16	8	8
NEO+PB	4	4	2	4	4	4	4	4	4	2	8	4	4	4	8
R-NEO+PB	1	2	1	1	1	2	2	1	4	4	2	2	1	2	4
C-NEO+PB	4	2	2	1	2	2	2	2	2	2	1	1	1	2	2
RC-NEO+PB	2	2	2	1	0.5	1	1	1	2	1	1	0.5	2	1	2

Tables S23 – S25: [a]The concentration range for PB was 0.125 – 8 μM and for PB in the combinations was 0.0625 – 4 μM. The MICs are given for NEO, R-NEO, C-NEO and RC-NEO in the combination with PB. [b]Reflects the MICs found after a 24 h removal of the drug or drug combination from day 14 cultures.

Table S26. Minimal bactericidal concentration (MBC, µM) with drugs alone and in combination with PB at day 1 & 14 of the resistance development assay.

Treatment	*A. baumannii* 19606	*K. pneumoniae* 0558	*P. aeruginosa* 0668
PB	1, (4)	4, (16)	4, (16)
NEO	8, (16)	4, (32)	16, (16)
R-NEO	64, (64)	32, (32)	>64, (>64)
C-NEO	32, (>64)	32, (64)	16, (32)
RC-NEO	64 (>64)	32, (64)	32, (32)
NEO+PB	4, (8)	2, (8)	16, (32)
R-NEO+PB	4, (16)	4, (4)	8, (16)
C-NEO+PB	8, (16)	2, (4)	4, (4)
RC-NEO+PB	4, (8)	4, (8)	16, (16)

Numbers in parentheses are MIC for day 14. The MICs were recorded after 24 h incubation with drugs in 96 well plates by OD600. For the minimal bactericidal concentration (MBC), the entire contents from the wells (100 µL) that showed > 95% growth inhibition where diluted two-fold in MHII broth and 200 µL was plated to nutrient agar plates (85 mm diameter) and incubated for 24 h at 37°C to allow recovery of bacterial colonies.

Table S27. Complete genotypic antibiotic resistance profile and aminoglycoside resistance profile for the CDC clinical isolate *K. pneumoniae* strain 0558.

		Others		Mono-substituted 2-deoxystreptamines			4,5-di-substituted 2-deoxystreptamines			4,6-di-substituted 2-deoxystreptamines						
**Strain/#**	**Resistance Genes**	**STR**	**SPT**	**APR**	**NEA**	**HYG**	**PAR**	**NEO**	**RIB**	**KAN**	**NET**	**AMK**	**GEN**	**TOB**	**SIS**	**PLZ**
**Kpn 0558**	*aac(3)-IId, aac(6')-Ib-cr, aadA1, aadA2, armA ARR-2, catA1, CTX-M-15, dfrA12, dfrA14, EMRD, ere(A), fosA5, KDEA, OXA-181, SHV-26, sul1, sul2, tet(A), tet(R)*	12.5	>50	>50	>50	>50	3.13	1.56	50	>50	>50	>50	>50	50	>50	50

Four main subclasses of aminoglycoside antibiotics: no deoxystreptamine (no DOS), monosubstituted 2-deoxystreptamines (mono 2-DOS), 4,5-disubstituted 2-deoxystreptamines (4,5-DOS), and 4,6-disubstituted 2-deoxystreptamines (4,6-DOS).[2] Spectinomycin is included in the above data as well although it is technically classified as an aminocyclitol antibiotic and differs from aminoglycosides in that it does not contain amino sugars or glycosidic bonds but is closely related in its mechanism of action.[3] The only aminoglycoside listed with no deoxystreptamine would be streptinomycin, which has a strepadine ring instead of a streptamine ring. Minimal inhibitory concentrations (MICs) were set up and performed as microbroth dilutions adhering to the Clinical Laboratory Standards Institute (CLSI) and all data values are reported in µM concentrations. For CDC bacterial isolates, resistance gene profiling was performed by the CDC and identified by analysis of whole genome sequence using the ResFinder database (last updated June 2, 2016 and accessed on October 25, 2016) and the CDC is in the process of updating the resistance genes for the clinical isolate bank. Additional information, as well as complete antibiotic profiles for the CDC isolates, can be found on the CDC website at https://wwwn.cdc.gov/ARIsolateBank/Panel/AllIsolate. Abbreviations: STR: streptomycin; SPT: spectinomycin; APR: apramycin; NEA: neamine; HYG: hygromycin; PAR: paromomycin; NEO: neomycin; RIB: ribostamycin; KAN: kanamycin; NET: netilmicin; AMK: amikacin; GEN: gentimicin; TOB: tobramycin; SIS: sisomicin; PLZ: plazomicin; Pstu: *Providencia stuartii*; Kpn: *Klebsiella pneumoniae*; Sseft: *Salmonella seftenberg;* Abau: *Acinetobacter baumannii*; Ecloa: *Enterobacter cloacae*; Paer: *Pseudomonas aeruginosa;* NR = None Reported, ND = No Data; CLSI: Clinical Laboratory Standards Institute.

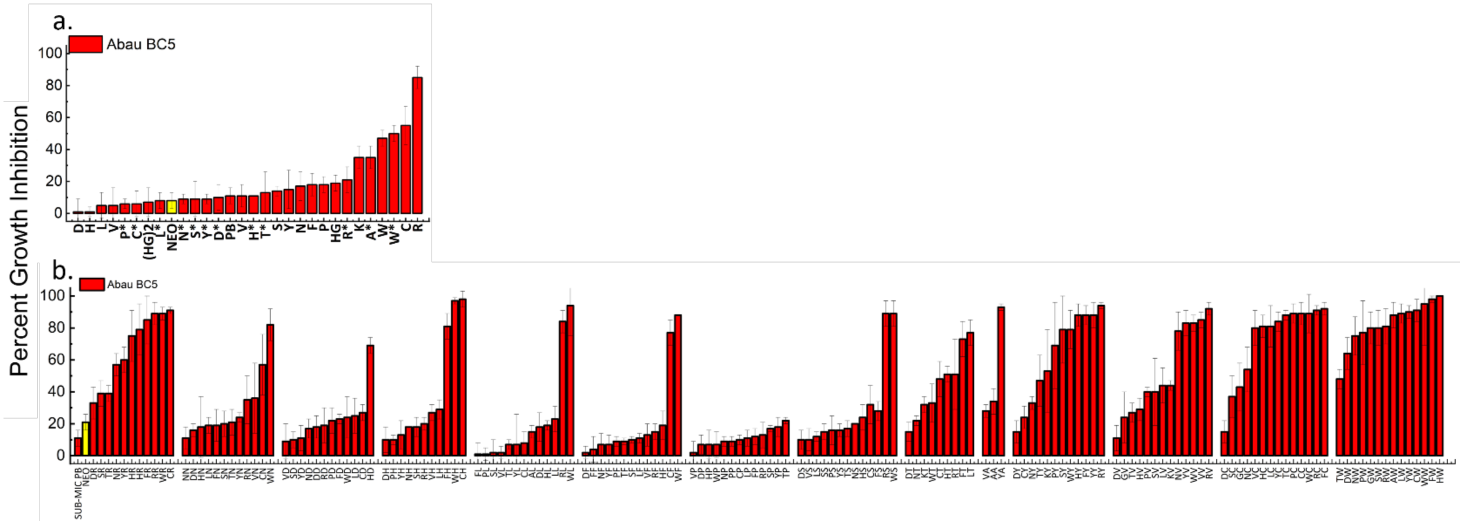

Figure S1. Percent growth inhibition in a single point synergy screen (SPSS) of a P-NEO library (5 μM) in combination with sub-MIC of polymyxin B (PB) against NEO[R] *A. baumannii* strain BC5. The sub-MIC of PB did not significantly affect growth after a 24 h incubation (≤20% growth inhibition). Neomycin linked with one amino acid (**a**, A-NEO) and with a β-alanine end group (marked with an asterisk*), neomycin linked to two amino acids (**b**, AA-NEO) Abbreviations: For the common amino acid adjacent to the amino acid and linked to NEO: R: Arginine, N: Asparagine, D: Aspartic acid, H: Histidine, L: Leucine, F: Phenylalanine, P: Proline, S: Serine, T: Threonine, A: Alanine, Y: Tyrosine, V: Valine, C: Cysteine, W: Tryptophan. Error bars represent the standard deviation of the mean of three replicates. The percent growth inhibition values are relative to growth control without PB, ≤20% growth inhibition is considered insignificant. Neomycin (NEO) was also screened at 5 μM (yellow bar) and served as a control. Conjugates which inhibited growth by ≥70% in combination with PB were used in checkerboard assays.

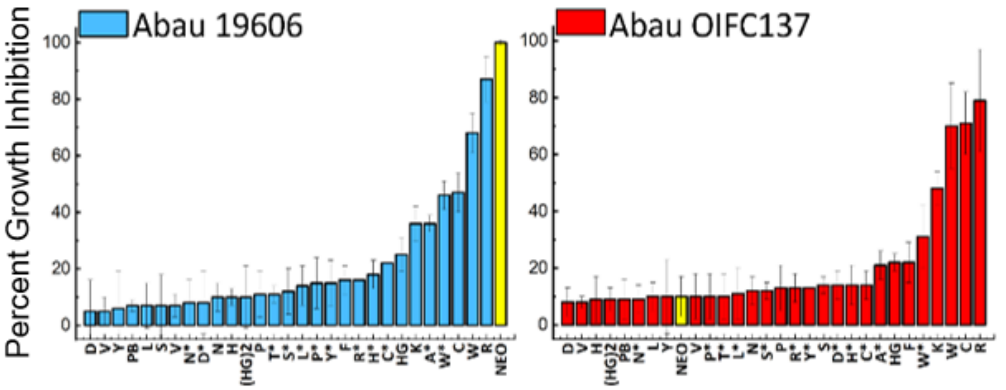

Figure S2. Percent growth inhibition in a single point synergy screen (SPSS) of a P-NEO library (5 μM) in combination with sub-MIC of polymyxin B (PB) against NEO[R] *A. baumannii* strains 19606 and OIFC137. The sub-MIC of PB did not significantly affect growth after a 24 h incubation (≤20% growth inhibition). Neomycin linked with one amino acid and with a β-alanine end group (marked with an asterisk*), neomycin linked to two amino acids (**b**, AA-NEO) Abbreviations: For the common amino acid adjacent to the amino acid and linked to NEO: R: Arginine, N: Asparagine, D: Aspartic acid, H: Histidine, L: Leucine, F: Phenylalanine, P: Proline, S: Serine, T: Threonine, A: Alanine, Y: Tyrosine, V: Valine, C: Cysteine, W: Tryptophan. Error bars represent the standard deviation of the mean of three replicates. The percent growth inhibition values are relative to growth control without PB, ≤20% growth inhibition is considered insignificant. Neomycin (NEO) was also screened at 5 μM (yellow bar) and served as a control. Conjugates which inhibited growth by ≥70% in combination with PB were used in checkerboard assays.

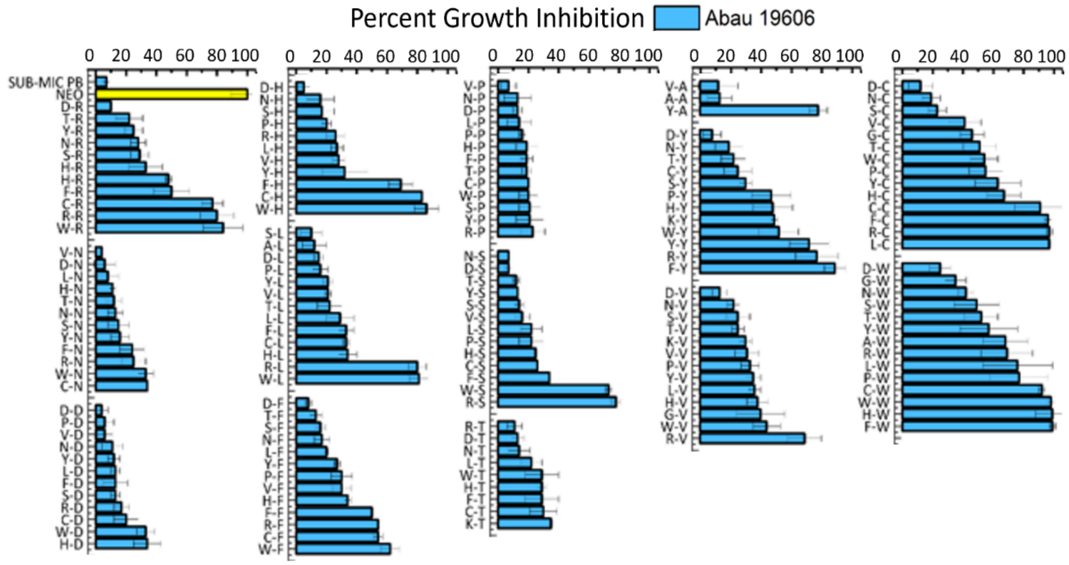

Figure S3. Percent growth inhibition in a single point synergy screen (SPSS) of a P-NEO library (5 μM) in combination with sub-MIC of polymyxin B (PB) against NEO[S] *A. baumannii* strain 19606. The sub-MIC of PB did not significantly affect growth after a 24 h incubation (≤20% growth inhibition). Neomycin linked with one amino acid and with a β-alanine end group (marked with an asterisk*), neomycin linked to two amino acids (**b**, AA-NEO) Abbreviations: For the common amino acid adjacent to the amino acid and linked to NEO: R: Arginine, N: Asparagine, D: Aspartic acid, H: Histidine, L: Leucine, F: Phenylalanine, P: Proline, S: Serine, T: Threonine, A: Alanine, Y: Tyrosine, V: Valine, C: Cysteine, W: Tryptophan. Error bars represent the standard deviation of the mean of three replicates. The percent growth inhibition values are relative to growth control without PB, ≤20% growth inhibition is considered insignificant. Neomycin (NEO) was also screened at 5 μM (yellow bar) and served as a control. Conjugates which inhibited growth by ≥70% in combination with PB were used in checkerboard assays.

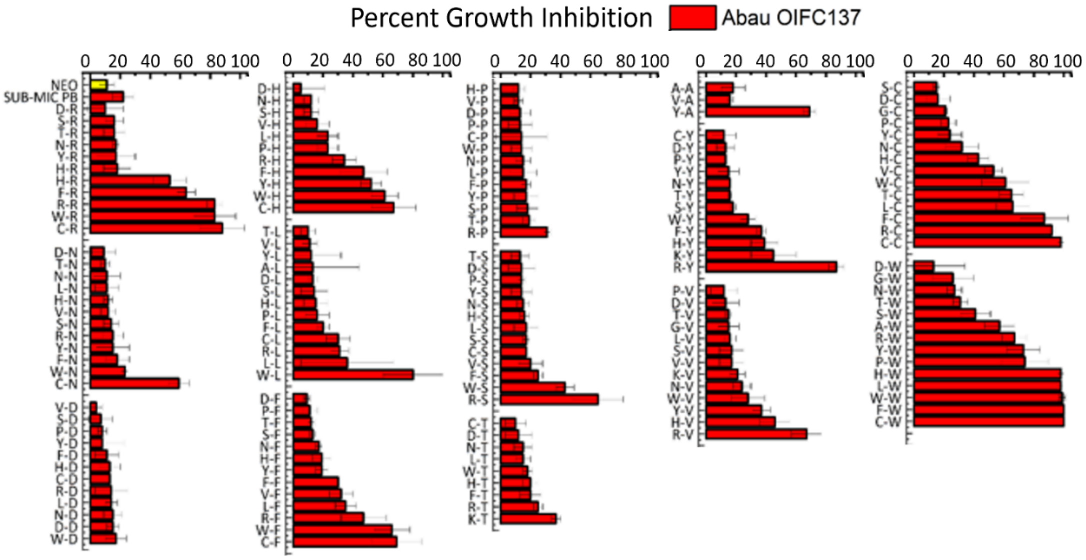

Figure S4. Percent growth inhibition in a single point synergy screen (SPSS) of a P-NEO library (5 μM) in combination with sub-MIC of polymyxin B (PB) against NEO[R] *A. baumannii* strain OIFC137. The sub-MIC of PB did not significantly affect growth after a 24 h incubation (≤20% growth inhibition). Neomycin linked with one amino acid and with a β-alanine end group (marked with an asterisk*), neomycin linked to two amino acids (**b**, AA-NEO) Abbreviations: For the common amino acid adjacent to the amino acid and linked to NEO: R: Arginine, N: Asparagine, D: Aspartic acid, H: Histidine, L: Leucine, F: Phenylalanine, P: Proline, S: Serine, T: Threonine, A: Alanine, Y: Tyrosine, V: Valine, C: Cysteine, W: Tryptophan. Error bars represent the standard deviation of the mean of three replicates. The percent growth inhibition values are relative to growth control without PB, ≤20% growth inhibition is considered insignificant. Neomycin (NEO) was also screened at 5 μM (yellow bar) and served as a control. Conjugates which inhibited growth by ≥70% in combination with PB were used in checkerboard assays.

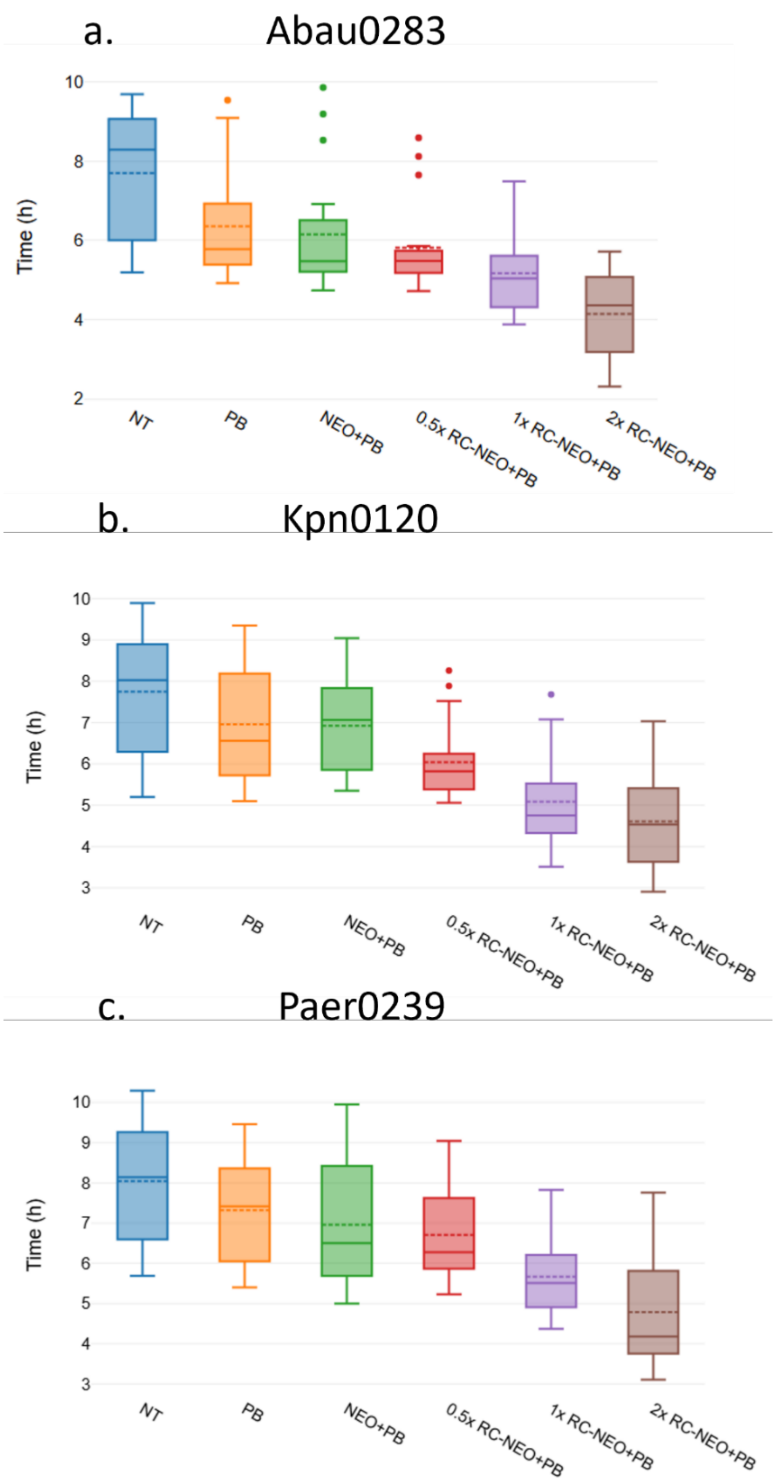

Figure S5. Box plots for time-kill data for *A. baumannii* 0283, *K. pneumoniae* 0120 and *P. aeruginosa* 0239.

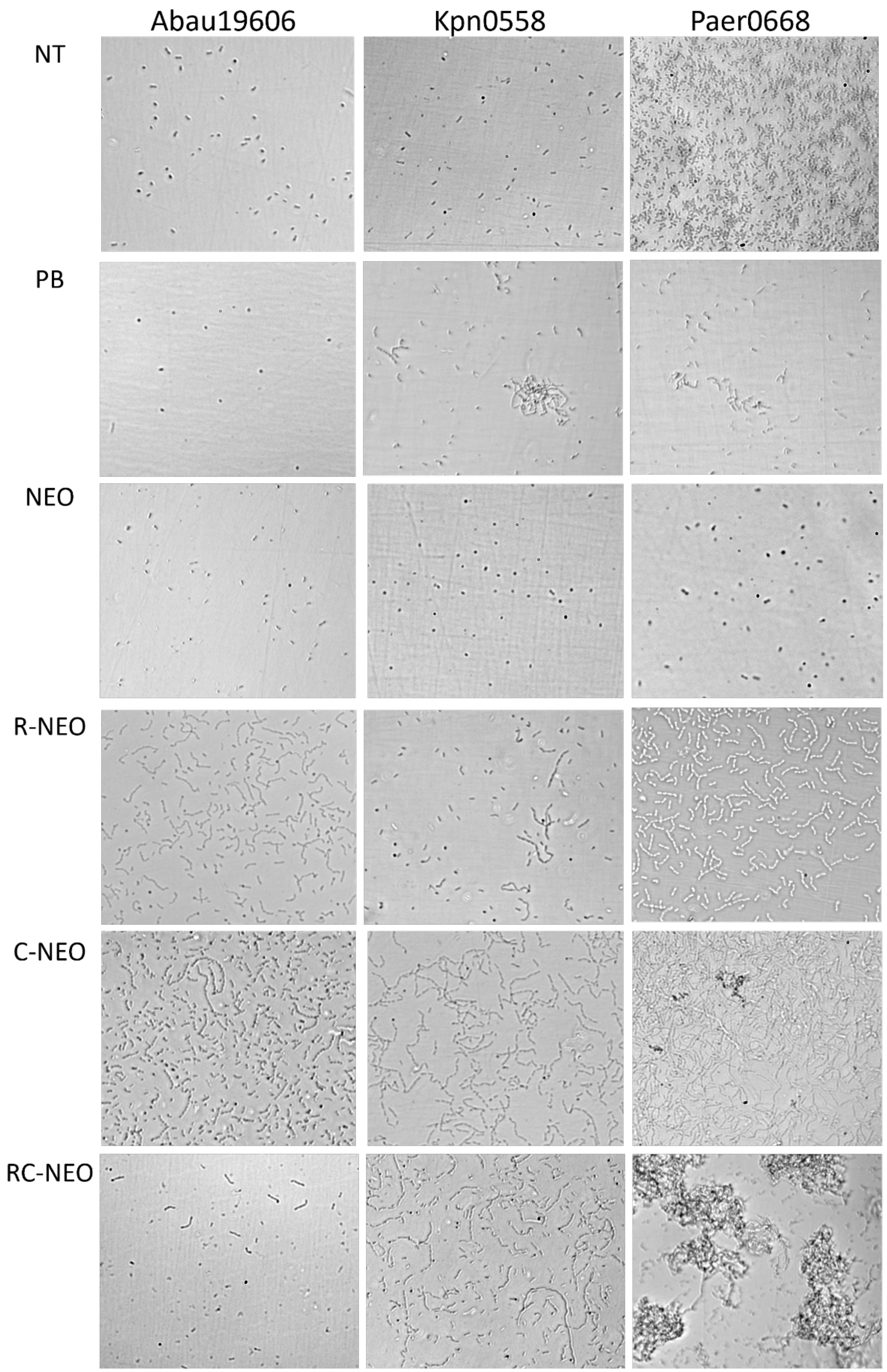

Figure 6S. Brightfield microscopy demonstrating changes in cell morphology causes by drugs or drug combinations in *A. baumannii*, *K. pneumoniae* and *P. aeruginosa* strains on day 1 of the resistance development assay.

(1) Jiang, L.; Watkins, D.; Jin, Y.; Gong, C.; King, A.; Washington, A. Z.; Green, K. D.; Garneau-Tsodikova, S.; Oyelere, A. K.; Arya, D. P. Rapid Synthesis, RNA Binding, and Antibacterial Screening of a Peptidic-Aminosugar (PA) Library. *ACS Chemical Biology* **2015**, *10* (5), 1278-1289. DOI: 10.1021/cb5010367 (acccessed 05/15).
(2) Krause, K. M.; Serio, A. W.; Kane, T. R.; Connolly, L. E. Aminoglycosides: An Overview. *Cold Spring Harb Perspect Med* **2016**, *6* (6). DOI: 10.1101/cshperspect.a027029 From NLM.
(3) Papich, M. G. Spectinomycin, Spectinomycin Dihydrochloride Pentahydrate. In *Saunders Handbook of Veterinary Drugs (Fourth Edition)*, Papich, M. G. Ed.; W.B. Saunders, 2016; pp 735-736.
